# Robots As Intentional Agents: Using Neuroscientific Methods to Make Robots Appear More Social

**DOI:** 10.3389/fpsyg.2017.01663

**Published:** 2017-10-04

**Authors:** Eva Wiese, Giorgio Metta, Agnieszka Wykowska

**Affiliations:** ^1^Department of Psychology, George Mason University, Fairfax, VA, United States; ^2^Istituto Italiano di Tecnologia, Genoa, Italy

**Keywords:** attribution of intentionality, mind perception, social robotics, human–robot interaction, social neuroscience

## Abstract

Robots are increasingly envisaged as our future cohabitants. However, while considerable progress has been made in recent years in terms of their technological realization, the ability of robots to interact with humans in an intuitive and social way is still quite limited. An important challenge for social robotics is to determine how to design robots that can perceive the user’s needs, feelings, and intentions, and adapt to users over a broad range of cognitive abilities. It is conceivable that if robots were able to adequately demonstrate these skills, humans would eventually accept them as social companions. We argue that the best way to achieve this is using a systematic experimental approach based on behavioral and physiological neuroscience methods such as motion/eye-tracking, electroencephalography, or functional near-infrared spectroscopy embedded in interactive human–robot paradigms. This approach requires understanding how humans interact with each other, how they perform tasks together and how they develop feelings of social connection over time, and using these insights to formulate design principles that make social robots attuned to the workings of the human brain. In this review, we put forward the argument that the likelihood of artificial agents being perceived as social companions can be increased by designing them in a way that they are perceived as intentional agents that activate areas in the human brain involved in social-cognitive processing. We first review literature related to social-cognitive processes and mechanisms involved in human–human interactions, and highlight the importance of perceiving others as intentional agents to activate these social brain areas. We then discuss how attribution of intentionality can positively affect human–robot interaction by (a) fostering feelings of social connection, empathy and prosociality, and by (b) enhancing performance on joint human–robot tasks. Lastly, we describe circumstances under which attribution of intentionality to robot agents might be disadvantageous, and discuss challenges associated with designing social robots that are inspired by neuroscientific principles.

## Introduction

Robots are becoming a vision for societies of the near future, partially due to a growing need for assistance beyond what is currently possible with a human workforce (Ward et al., 2011). Robots can assist humans in a wide spectrum of domains (Tapus and Matarić, 2006; Cabibihan et al., 2013) that are not necessarily limited to the *three d’s* (dirty, dangerous, dull) of robotics, where robots are envisaged to assist humans during tasks that are hazardous, repetitive, or prone to errors (Takayama et al., 2008). On the contrary, there is a plethora of other domains where robots can (and perhaps should be) deployed, including entertainment, teaching, and health care: Pet robots like Paro (Shibata et al., 2001), or AIBO (developed by Sony^1^, see also Fujita and Kitano, 1998) or the huggable pillow-phone robot, Hugvie (Yamazaki et al., 2016) are used for elderly patients to reduce loneliness, increase social communicativeness, or improve cognitive performance (Tapus et al., 2007; Birks et al., 2016), and have positive effects on mood, emotional expressiveness and social bonding among dementia patients (Martin et al., 2013; Birks et al., 2016). In addition to their applicability for elderly patients (Wada et al., 2005; Wada and Shibata, 2006), social robots (a) are used in therapeutical interventions for children with autism spectrum disorder to help practice social skills, such as joint attention, turn-taking or emotion understanding (Dautenhahn, 2003; Robins et al., 2005; Ricks and Colton, 2010; Scassellati et al., 2012; Tapus et al., 2012; Cabibihan et al., 2013; Anzalone et al., 2014; Bekele et al., 2014; Kajopoulos et al., 2015; Warren et al., 2015), and (b) improve outcomes for patients with sensorimotor impairments during rehabilitation (Hogan and Krebs, 2004; Prange et al., 2006; Basteris et al., 2014). Outside the clinical context, social robots foster collaboration in the workplace (Hinds et al., 2004), improve learning (Mubin et al., 2013), and problem solving (Chang et al., 2010; Castledine and Chalmers, 2011; Kory and Breazeal, 2014), and deepen students’ understanding of mathematics and science in the classroom (Fernandes et al., 2006; Church et al., 2010). They also facilitate activities in daily lives, either as friendly companions at home (Kidd and Breazeal, 2008; Graf et al., 2009), or as assistants in supermarkets and airports (Triebel et al., 2016).

Despite this number of positive examples where robots support and assist their human counterparts in everyday life, general attitudes toward robots are not always positive (Flandorfer, 2012). In fact, the general public can be quite skeptical with respect to the introduction of robot assistants in everyday life (Bartneck and Reichenbach, 2005), especially when aspects like signing over decision-making or control to the robot are at stake (Scopelliti et al., 2005). Pop culture, myths and novels in western cultures also often depict robots or artificial agents as a threat to humanity (Kaplan, 2004). As a result, users might be worried about violations of their privacy or about becoming dependent on robot technology (Cortellessa et al., 2008), and particularly elderly individuals might be concerned about integrating robots into their home environment (Scopelliti et al., 2005). Concerns have also been raised regarding the potential of robots to contribute to social isolation and deprivation of human contact (Sharkey, 2008), and assistive robots are at risk of becoming stigmatized by the media as tools for lonely, old and dependent users. In line with this assumption, elderly individuals are reluctant to accept robots as social companions for themselves, although they acknowledge their potential benefits for other user groups (Neven, 2010).

**FIGURE 1:**
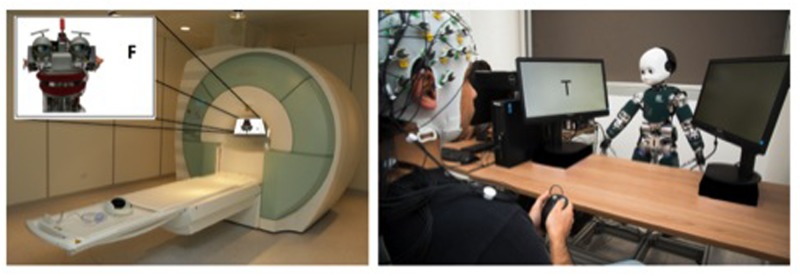
Investigation of human-robot interaction with the use of neuroscientific methods. The image on the left illustrates the setup of an fMRI experiment measuring changes in blood flow in social brain areas during a joint attention task with the robot EDDIE (designed by Technical University of Munich). Participants are asked to respond as fast and accurately as possible to the identity of a target letter (“F” vs. “T”) that is either looked at nor not looked at by EDDIE. Changes in activation in social brain areas can be captured with a high spatial resolution, but no natural interactivity with the robot can be achieved (i.e., interaction needs to be imagined: *offline social cognition*). The image on the right shows a setup where neural processes associated with joint attention are examined using EEG and eye-tracking during interactions with the robot iCub (designed at the Istituto Italiano di Technologia by Metta et al., 2008). Similar to the previous example, participants are asked to react to the identity of a target letter (“T” vs. “V”) that is either looked at or not looked at by iCub. Mechanisms of joint attention can be captured with high temporal resolution during relatively natural interactions (i.e., *online social cognition*). Written informed consent has been obtained for publication of the identifiable image on the right.

Overall, these studies reveal that humans can be willing to accept social robots in some contexts but might be reluctant to do so in others. In consequence, research in social robotics needs to determine not only how to design robots that optimally support stakeholders with different cognitive and technical abilities, but also which features robots need to have to in order to be accepted as social companions that understand our needs, feelings and intentions, and can share valuable experiences with humans. One problem with the current state of social robotics research is that it often lacks systematicity, and in effect, specifications of particular features that facilitate treating robots as social companions are not sufficiently addressed. We suggest addressing this issue by using behavioral and physiological neuroscience methods (i.e., eye-tracking, EEG, fNIRS, fMRI) in robotics research with the goal of objectively measuring how humans react to robot agents, how they perform tasks with robots and how they develop mutual understanding and social engagement over time. In this context, we note that each method has advantages and disadvantages, and is suitable for specific questions but not others (for examples, see **Table 1** and **Figure 1**). Insights gained from applying these methods can then be used to formulate design principles for social robots that are attuned to the workings of the human brain. In particular, we argue that if robots are to be treated as social companions, they should evoke mechanisms of social cognition in the brain that are typically activated when humans interact with other humans, such as joint attention (Moore and Dunham, 1995; Baron-Cohen, 1997), spatial perspective-taking (Tversky and Hard, 2009; Zwickel, 2009; Samson et al., 2010), action understanding (Gallese et al., 1996; Rizzolatti and Craighero, 2004; Brass et al., 2007), turn-taking (Knapp et al., 2013), and mentalizing (Baron-Cohen, 1997; Frith and Frith, 2006a).

**Table 1:** Advantages and disadvantages of measures used to investigate human–robot interaction, together with example questions that can be best addressed with the respective measure; ERP, event related potential; PSP, postsynaptic potential; fMRI, functional magnetic resonance imaging; fNIRS, functional near infrared spectroscopy; TDS, transcranial doppler sonography; BF, blood flow.

Method	Advantages	Disadvantages	Questions (examples)
**Subjective measures**	Explicit processes	Subjective measures	Traits
Likert scales	Inexpensive	Social acceptability bias	Attitudes
Implicit association	Easy-to-implement	Disrupts natural interaction	Acceptance
Interviews		No implicit processes	Judgments
		No performance measure	Likability
			Classification/stereotyping
**Performance measures**	Objective measures	Disrupts natural interaction	Effectiveness/efficiency
Reaction times	Implicit/explicit processes	Needs specified goals	Competition
Error rates	Inexpensive	Indirect neural measure	Distraction
	Easy-to-implement		Cognitive load
			Social attention
			Joint action
			Search and rescue
**Behavioral measures**	Objective measures	Some discomfort	Free exploration (mobile)
Eye tracking	Implicit processes	Feeling of unnaturalness	Natural interaction (mobile)
Motion tracking	Relatively inexpensive	Indirect neural measure	Social attention (mobile)
	Exploratory research	Not suitable for everyone	Social dynamics
	Non-disruptive		Preferences
			Stress
			Cognitive load
			Movement kinematics
**Physiological measures**	Objective measures	Not specific in terms of cognitive processes	Stress
Heart rate	Implicit processes	Indirect neural measure	Alertness
Skin conductance	Relatively inexpensive	Low temporal resolution	Engagement
Respiratory rate	Non-disruptive		Cognitive load
**Electroencephalography**	Objective measures	Some discomfort	Engagement
ERPs	Implicit processes	Feeling of unnaturalness	Social reward
(Time-) frequency	Relatively inexpensive	Timely to set-up	Task monitoring
	Non-disruptive	Bound to laboratory setting	Error processing
	Direct neural measure (PSP)	Low spatial resolution	Entrainment
	High temporal resolution	Movement/other artifacts	Conflict processing
	Source localization possible		Social attention
			Joint action
			Violation of expectation
**Neuroimaging**	Objective measures	Some discomfort	Social reward
fMRI	Implicit processes	Feeling of unnaturalness	Social attention
fNIRS	Non-disruptive	Expensive	Bonding
TDS	Direct neural measure (BF)	Low temporal resolution	Empathy
	High spatial resolution	Movement/other artifacts	Imitation
	Source localization possible		Anthropomorphism
			Mind perception


But how can we make robots and other automated agents appear social? Research suggests that the two most important aspects for artificial agents to appear social are human-like appearance and behavior (Tapus and Matarić, 2006; Waytz et al., 2010b; Wykowska et al., 2016), with behavior probably being even more critical than appearance (a speculation that needs to be tested empirically). The effectiveness of behavior in inducing perceptions of humanness can be seen in Sci-Fi movies, where agents with not very human-like appearance like C-3PO, Wall-E, or Baymax can be perceived as social entities that evoke sympathy or affinity because their behavior is so human-like. On the other hand, human-looking agents like Data (‘Star Trek’) or Terminator (‘Terminator’) can evoke a sense of oddness or discomfort when they show mechanistic behavior. We suggest that research should build upon these observations and investigate (a) which physical and behavioral agent features are associated with humanness and are therefore able to make artificial entities appear social, and (b) how perceiving artificial agents as social entities affects attitudes, acceptance and performance in human–robot interaction. In order to accomplish that, it is useful to first understand the neural and cognitive underpinnings of social cognition in human–human interaction and then examine whether similar mechanisms can be activated in human–robot interaction. The ultimate goal is to create robots that are human-like enough to evoke mechanisms of social cognition in human interaction partners, and to achieve this with the use of neuroscientific and psychological methods.

This review focuses on humanoid robots (as opposed to robots with other non-humanoid shapes) for the following reasons: first, the goal of this review is to understand social interactions between humans and robots that live in shared environments. These environments are typically designed to match human movement and cognitive capabilities (in terms of physical space, ergonomics, or interfaces). Robots that are supposed to act as social interaction partners in the future need to fit in these human-attuned environments by emulating human form and cognition. For example, a legged service robot at a restaurant would be able to step over obstacles with which a wheeled robot might have troubles. Similarly, a robot of human-like width and height would be able to move around in human environments better than a larger robot. Human shape also allows the robot to communicate internal states like emotions (i.e., via facial expression or body posture) or intentions (i.e., via social cues like gestures or changes in gaze direction) in a natural way. Second, robots with a humanoid appearance have the potential to provide a number of desired functions within a single platform (i.e., service, learning, companionship), which allows for a more general and flexible use than more specialized platforms without human features. For example, in a home environment, a humanoid robot can manipulate kitchen utensils and appliances to cook (oven, fridge, dishwasher, etc.). The same robot can press buttons and control light settings, switch the television on and off, serve food, and utilize all tools that are already available at home, simplifying the humanoid robots’ deployment and increasing their usefulness, without substantially modifying the human environment. Lastly, the goal of this review is to advocate for the integration of behavioral and physiological neuroscience methods in the design and evaluation of social robots able to engage in social interactions, which requires robot platforms that are human-like enough to activate mechanisms in the human brain in a fashion similar to human interaction partners. Since many social-cognitive brain mechanisms are sensitive to human appearance and behavior (see “Observing Intentional Agents Activates Social Brain Areas”), humanoid robot designs are the most promising, but not necessarily the only avenue to accomplish this goal (for research on animal-like and fictional robot designs, see for instance, Shibata et al., 2001 or Kozima and Nakagawa, 2007). However, we acknowledge that for more specific and focused applications, other robot designs can be more suitable (Fujita and Kitano, 1998; Johnson and Demiris, 2005).

In this review, we argue that one of the main factors that contributes to robots being treated as social entities is their ability to be perceived as intentional^2^ beings with a mind (see “Can Robots be Perceived as Intentional Agents?”), so that they activate brain areas involved in social-cognitive processes in a similar way as human interaction partners do (see “Observing Intentional Agents Activates Social Brain Areas”). Since intentionality is a feature that can be ascribed to non-human agents or withdrawn from human agents (Gray et al., 2007), it is important to understand the principles underlying the attribution of intentionality to others, and to examine its effects on attitudes, acceptance and performance in human–human and human–robot interaction (see “Effects of Mind Perception on Attitudes and Performance in HRI”). The ultimate goal is to design social robots that trigger attributions of intentionality with a high likelihood and activate social-cognitive areas in the human brain (see “Designing Robots as Intentional Agents,” for examples of robot designs that are in line with neuroscientific models of the social brain).

## Can Robots Be Perceived As Intentional Agents?

In human–human interactions, we activate brain areas responsible for social-cognitive processing and make inferences about what others think, feel and intend based on observing their behavior (Frith and Frith, 2006a,b). However, before we usually make inferences about intentions or emotions, we need to perceive others as intentional beings, with the general ability of having internal states (i.e., *mind perception*; Gray et al., 2007). Attributing internal states in social interactions is the default mode for human agents, but this might not automatically happen during interactions with artificial agents like Siri, Waymo, or Jibo^3^ due to their ambiguous mind status. As a result, human brain areas specialized in processing inputs of intentional agents might not be sufficiently activated when interacting with robot agents, which can potentially have negative consequences on attitudes and performance in human–robot interactions. We suggest that this issue should be addressed in social robotics by incorporating neuroscientific methods in the engineering design cycle, with the goal of designing robots that activate social brain areas in a similar manner as human interaction partners do. Robots that are attuned to the human cognitive system have the potential to make human–robot interaction more intuitive, and can positively affect acceptance and performance within human–robot teams.

Luckily for human–robot interaction, perceiving mind is not exclusive to agents that actually have a mind, but can also be triggered by agents who are not believed to have a mind (i.e., robots, avatars, self-driving cars) or agents with ambiguous mind status (i.e., animals; Gray et al., 2007). Mind is in the eye of the beholder, which means that it can be ascribed to others or denied, based on cognitive or motivational features associated with the perceiver, as well as physical and behavioral features of the perceived agent (Waytz et al., 2010b). For instance, being in need of social connection or lacking system-specific knowledge has been shown to increase the likelihood that human characteristics like ‘having a mind’ are ascribed to non-human agents (i.e., *anthropomorphism*; Rosset, 2008; Hackel et al., 2014), while feeling socially rejected or witnessing harmful acts being done to others by human beings decreases the extent to which mind is perceived in them (i.e., *dehumanization*; Epley et al., 2007; Bastian and Haslam, 2010; Waytz et al., 2010a). The human tendency to anthropomorphize others is so strong that some of us readily perceive craters on the moon as the ‘man in the moon,’ burnt areas on a toast as ‘Jesus,’ or the front of a car as ‘having a face,’ and are not surprised when Tom Hanks becomes friends with the volleyball Wilson (‘*Cast Away’*), or when Joaquin Phoenix falls in love with his virtual agent Samantha (‘*Her*’).

In line with these observations, psychological research has shown that anthropomorphism, and specifically mind perception, are highly automatic processes that activate social areas in the human brain in a bottom–up fashion (Gao et al., 2010; Looser and Wheatley, 2010; Wheatley et al., 2011; Schein and Gray, 2015), triggered by human-like facial features (Maurer et al., 2002; Looser and Wheatley, 2010; Balas and Tonsager, 2014; Schein and Gray, 2015; Deska et al., 2016), or biological motion (Castelli et al., 2000). Due to the automatic nature of mind perception, intentional agents can be differentiated from non-intentional agents within a few 100 ms (Wheatley et al., 2011; Looser et al., 2013), and even just passively viewing stimuli that trigger mind perception is sufficient to induce activation in a wide range of brain regions implicated in social cognition (Wagner et al., 2011).

Using the anthropomorphic model when making inferences about the behavior of non-human entities makes sense given that we are experts in what it means to be human, but have no phenomenological knowledge about what it means to be non-human (Nagel, 1974; Gould, 1998). Thus, when we interact with entities for which we lack specific knowledge, we commonly choose the ‘human’ model to predict their behavior, such as blaming God for events that we cannot explain or thinking that computers want to sabotage us when they simply start to malfunction (Rosset, 2008). Once the human model is activated, we can use it to infer particular intentions behind observed actions (i.e., *mentalizing*) or to reason about emotional states underlying facial expressions or changes in body language (i.e., *empathizing*). We do this by imagining what we would intend or feel if we were in a comparable situation (Buccino et al., 2001; Umilta et al., 2001; Rizzolatti and Craighero, 2004), which gives us immediate phenomenological access to the internal states of others. Despite the advantage of being able to reason about their internal states, automatically activating the anthropomorphic model when interacting with robots could also have negative consequences when it leads to incorrect predictions because the behavioral repertoire of the robot does not perfectly overlap with human behavior (Bisio et al., 2014), or when it potentially induces a cognitive conflict because certain robot features trigger mind perception (i.e., appearance), while others hinder mind perception (i.e., motion; Chaminade et al., 2007; Saygin et al., 2012; see “Negative Effects of Mind Perception in Social Interactions”). For a detailed discussion of costs and benefits associated with anthropomorphism in human–robot interaction, please also see (Złotowski et al., 2015).

In sum, these studies suggest that non-human agents have the potential to trigger mind perception, as long as they display observable signs of intentionality, such as human-like appearance and/or behavior. In this review, we argue that mind perception has the potential to positively affect human–robot interaction by (a) activating the social brain network involved in action understanding and mentalizing, (b) enhancing feelings of social connection, empathy and prosociality, and (c) fostering performance during joint action tasks. However, we also discuss circumstances in which mind perception might be disadvantageous for human–robot interaction, and suggest robot design features that allow humans to flexibly activate and deactivate the ‘human’ model when interacting with robot agents.

## Observing Intentional Agents Activates Social Brain Areas

**FIGURE 2:**
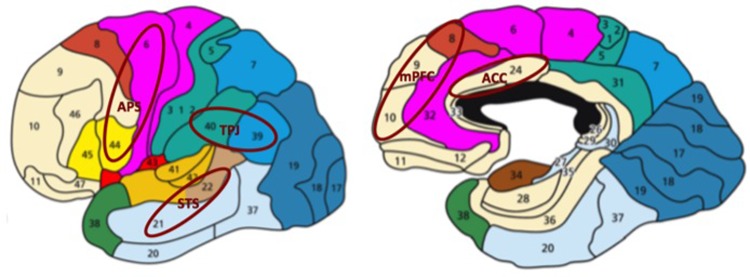
Social brain network consisting of the action perception system (APS, mainly brodmann areas 6, 44, but also 4 and 40), superior temporal sulcus (STS; brodmann areas 21, 22), temporo-parietal junction (TPJ; brodmann areas 39,40), medial prefrontal cortex (mPFC; brodmann areas 8, 9, 10, 32) and anterior cingulate cortex (ACC; brodmann area 24). APS and STS detect biological motion and make inferences about low-level action goals from observed behavior. TPJ and mPFC are involved in mentalizing about high-level action goals and stable person features. ACC is associated with the attribution of mental states to non-human entities. The image has been modified (the original image was retrieved from: http://2.bp.blogspot.com/-SE4Yb_SRjdw/T6rNRgvRedI/AAAAAAAAAA0/FaU50ZemOCY/s1600/brodmann.png).

In order to successfully interact with others, we need to understand and predict their behavior (see “Performing Actions Together: Action Understanding and Joint Action”), and be able to make inferences about their intentions and emotions (see “Making Inferences about Internal States: Mentalizing and Empathizing”). The human brain is highly specialized in understanding the behaviors and internal states of others, and contains areas that are specifically activated when we interact with other social entities (i.e., *social brain*; Adolphs, 2009). Understanding actions is subserved by frontoparietal networks of the action-perception system (APS), while reasoning about internal states activates the temporo-parietal junction (TPJ), as well as prefrontal areas like the medial prefrontal cortex (mPFC), and anterior cingulate cortex (ACC; Brothers, 2002; Saxe and Kanwisher, 2003; Amodio and Frith, 2006; Adolphs, 2009; van Overwalle, 2009; Saygin et al., 2012; see **Figure 2**). Activation within the social brain network is predictive of how much we like others, how strongly we empathize with them, and how well we understand their actions (Ames et al., 2008; Cikara et al., 2011; Gutsell and Inzlicht, 2012), and can therefore be used as a proxy to estimate the degree of socialness that is ascribed to others. Although non-human agents can generally activate the social brain network, the strength of activation depends on the degree to which they are perceived as human-like entities with a mind (Blakemore and Decety, 2001; Gallese et al., 2004; Chaminade et al., 2007). The following sections describe the social brain network in more detail and discuss whether social robots can activate these brain areas, and if so, under which conditions.

### Performing Actions Together: Action Understanding and Joint Action

One key mechanism in social interactions is the ability to understand the actions of others, that is: being able to tell what sort of action is executed, and based on what kind of intention. Action understanding in the primate brain is based on shared representations that are activated both when an action is executed and when a similar action is observed in others (i.e., *resonance*; Gallese et al., 1996; Decety and Grèzes, 1999). Observing the actions of others facilitates the execution of a similar action (i.e., motor imitation), and hinders the execution of a different action (i.e., motor interference), since both action observation and execution activate the same neural network (Kilner et al., 2003; Oztop et al., 2005; Press et al., 2005). Imitation/interference effects are observed, for instance, when participants perform continuous unidirectional arm movements while observing continuous arm movements in the same/orthogonal direction or when being asked to perform an opening/closing gesture with their hand while observing opening/closing gestures in others (Kilner et al., 2003; Oztop et al., 2005; Press et al., 2005). Shared representations are also essential for performing joint actions, where two or more individuals coordinate their actions in time and space to achieve a shared action goal (Sebanz et al., 2005; Sebanz and Knoblich, 2009). For instance, when performing an action coordination task with another person (e.g., to cause a moving circle to overlap with a moving dot), we need to represent our own action (e.g., accelerating the circle) together with the action the other person is performing (e.g., slowing down the moving circle) in order to accomplish a shared action goal (e.g., establish overlap between the circle and the dot; Knoblich and Jordan, 2003). Performing a task together with another person also impacts action planning (Sebanz et al., 2006), and action monitoring (van Schie et al., 2004), which provides further support for the involvement of shared representations in the execution of joint actions.

In the primate brain, action understanding and execution activate the APS, including temporal areas like the posterior superior temporal sulcus (pSTS), involved in processing biological motion, as well as frontoparietal areas like the inferior parietal cortex and ventral premotor cortex (IPC and vPMC), responsible for inferring the intentions underlying observed actions (Saygin et al., 2004; Becchio et al., 2006; Pobric and Hamilton, 2006; Grafton and Hamilton, 2007; Saygin, 2007). In non-human primates, the IPC and vPMC are known to contain mirror neurons that fire both during action observation and execution, and infer intentions by simulating the action outcome as if the observer was executing the actions himself (Gallese et al., 1996, 2004; Keysers and Perrett, 2004; Rizzolatti and Craighero, 2004; Iacoboni, 2005). Although there is agreement that action understanding in humans is also based on the principles of resonance (Umilta et al., 2001; Kilner et al., 2003; Oztop et al., 2005; Press et al., 2005), the particular role of mirror neurons in this process still needs to be determined (Dinstein et al., 2007; Chong et al., 2008; Kilner et al., 2009; Mukamel et al., 2010; Saygin et al., 2012).

Given the importance of action understanding in human–robot interaction, it is essential to examine whether activation within the APS is exclusive to human agents or whether robotic agents can also activate this network. Robots were initially not assumed to activate the APS due to the fact that activation in this network is sensitive to the observation of biological motion and intentional behavior. In line with this assumption, initial studies on action understanding in human–robot interaction were not able to show motor resonance for the observation of robot actions (Kilner et al., 2003) or at least to a significantly smaller degree than for the observation of human agents (Oztop et al., 2005; Press et al., 2005; Oberman et al., 2007). Follow-up studies consistently showed that motor resonance can be induced by robot agents, but that its degree seems to depend on features like physical appearance (Chaminade et al., 2007; Kupferberg et al., 2012), motion kinematics (Bisio et al., 2014), or visibility of the full body (Chaminade and Cheng, 2009). In contrast, beliefs regarding the humanness of the observed agent did not have an impact on the presence or absence of motor resonance (Press et al., 2006). Yet another set of studies showed that motor resonance during interactions with robot agents can even reach levels comparable to human agents, however, only when participants were explicitly instructed to pay attention to their actions (Gazzola et al., 2007; Cross et al., 2011; Wykowska et al., 2014a), or when given additional time to familiarize themselves with the robots’ actions (Press et al., 2007). These findings suggest that participants naturally pay more attention to human actions than robot actions, with the consequence that brain areas involved in action understanding and prediction might be under-activated during interactions with robots. However, this effect can be reverted if participants are encouraged to process robot actions at a sufficient level of detail, either via instruction or via increased familiarization time.

Altogether, these studies suggest that robots have the potential to activate the human APS, at the very least in a reduced fashion, but under certain conditions even to a similar degree as human interaction partners. The degree of activation in APS depends on physical factors, such as the appearance or kinematic profile of a robot agent, as well as cognitive factors, such as one’s willingness to reason about a robot’s intentionality or the level of expertise one has with a particular robotic system. This means that low-level mechanisms of social cognition are not specifically sensitive to the identity of an interaction partner, and can be activated by robot agents as long as their actions map onto the human motor repertoire, and people are motivated to pay attention to them.

### Making Inferences about Internal States: Mentalizing and Empathizing

When navigating social environments, we need to understand how others feel (i.e., *empathizing*; Baron-Cohen, 2005; Singer, 2006), and what they intend to do (i.e., *mentalizing*: Frith and Frith, 2003, 2006a). Similar to joint action, empathizing and mentalizing are based on shared representations that allow us to infer the emotions and intentions of others by simulating what we would feel or intend in a comparable situation (i.e., represent the behavior of others in our own reference frame; de Guzman et al., 2016; Steinbeis, 2016). In terms of empathizing, seeing or imagining the emotional states of others automatically activates similar states in the observer, thereby creating a shared representation at the neural and physiological level (Preston and de Waal, 2002). For instance, receiving a painful stimulus and observing the stimulus being presented to others activates similar brain areas, involving the anterior insula, rostral ACC, brain stem, and cerebellum (Singer et al., 2004). Similarly, smelling disgusting odors and seeing faces disgusted by the presentation of the same odors activates shared representations in the anterior insula (Wicker et al., 2003), and being touched and observing someone else being touched at the same parts of the body activates similar areas within the secondary somatosensory cortex (SII; Keysers et al., 2004).

When studying mentalizing, researchers typically present participants with stories that involve false-belief manipulations (Wimmer and Perner, 1983; Baron-Cohen et al., 1985) and require them to (a) take the perspective of others in order to understand whether and how their representation of the situation differs from their own (Epley et al., 2004; Epley and Caruso, 2009), (b) make inferences about what others are interested in based on non-verbal cues like gaze direction (Friesen and Kingstone, 1998), and (c) reason about how others currently feel based on facial expressions or body postures (Baron-Cohen, 2005; Singer, 2006). In the human brain, processes related to mentalizing are subserved by a distributed network consisting of temporal areas like the TPJ, as well as prefrontal areas like the mPFC and ACC (Ruby and Decety, 2001; Chaminade and Decety, 2002; Farrer et al., 2003; Grèzes et al., 2004, 2006; van Overwalle, 2009). Bilateral TPJ is involved in inferring intentions based on sensory input (Gallagher et al., 2000; Ruby and Decety, 2001; Chaminade and Decety, 2002; Saxe and Kanwisher, 2003; Grèzes et al., 2004, 2006; Ohnishi et al., 2004; Perner et al., 2006; Saxe and Powell, 2006), and allows differentiating self from other intentions via perspective-taking (Ruby and Decety, 2001; Chaminade and Decety, 2002; Farrer et al., 2003; van Overwalle, 2009). Although both sides of the TPJ have basic mentalizing and perspective-taking abilities, expertise regarding these functions seems to be lateralized, with the left side being more specialized on perspective-taking (Samson et al., 2004), and the right side being more involved in mentalizing (Gallagher et al., 2002; Frith and Frith, 2003; Saxe and Kanwisher, 2003; Saxe and Wexler, 2005; Costa et al., 2008). Activation within left TPJ is also associated with attributions of humanness (Chaminade et al., 2007; Zink et al., 2011) and intentionality (Perner et al., 2006) to non-human agents, and gray matter volume in left TPJ has been shown to be a reliable predictor for individual differences in anthropomorphizing non-human agents (Cullen et al., 2014). Right TPJ is specialized on inferring intentions underlying observed human behavior, and shows stronger activation for intentional than non-intentional or random actions (Gallagher et al., 2002; Cavanna and Trimble, 2006; Krach et al., 2008; Chaminade et al., 2012). In addition to its involvement in mentalizing, the TPJ also serves as a convergence point for processing social and non-social information (Mitchell, 2008; Scholz et al., 2009; Chang et al., 2013; Krall et al., 2015, 2016).

When we make inferences about the internal states of others, it is essential to incorporate knowledge about their dispositions and preferences into the mentalizing process, in particular in long-term interactions (van Overwalle, 2009). This requires the ability to represent behaviors over a long period of time, across different circumstances and with different social partners, and is associated with activation in the mPFC (Frith and Frith, 2001; Decety and Chaminade, 2003; Gallagher and Frith, 2003; Amodio and Frith, 2006). Neurons in the mPFC have the ability to discharge over extended periods of time and across different events (Wood and Grafman, 2003; Huey et al., 2006), and their activation is positively correlated to the degree of background knowledge we have about another person (Saxe and Wexler, 2005). The ventral mPFC is associated with reasoning about the emotional states of others (Hynes et al., 2006; Vollm et al., 2006), while the dorsal mPFC is more recruited during triadic interactions involving two agents and one object of interest (Brass et al., 2005; Jackson et al., 2006; Mitchell et al., 2006). Due to a high degree of interconnectivity with other brain areas, the mPFC can process a wealth of neural input and is capable of implementing abstract inferences regarding interpersonal information (Leslie et al., 2004; Amodio and Frith, 2006). Similar to the TPJ, the mPFC is more strongly activated by agents who are believed to have a mind (Krach et al., 2008; Riedl et al., 2014).

Perceiving others as intentional entities is particularly associated with activation in the ACC, a cortical midline structure extending from the genu to the corpus callosum (Barch et al., 2001). The anterior ACC is activated when we attribute internal states to others, and responds more strongly during interactions with intentional agents versus non-intentional agents (Gallagher et al., 2002), as well as during interactions that require real-time mentalizing rather than retrospective inferences about mental states based on stories or images (Gallagher et al., 2000; McCabe et al., 2001). In addition, the dorsal ACC is involved in processing uncertainty, while the ventral ACC is responsible for monitoring emotions in self and others (Bush et al., 2000; Barch et al., 2001; Critchley et al., 2003; Nomura et al., 2003; Amodio and Frith, 2006). Similar to the mPFC, the ACC is highly interconnected with other brain areas and plays an integrative role in both social and non-social cognitive processes (Allman et al., 2001).

In sum, these studies show that activation in brain areas related to empathizing and mentalizing are modulated by the degree to which interaction partners are perceived to have a mind, with stronger activation for intentional agents (i.e., humans) compared to non-intentional agents (i.e., robots; Leyens et al., 2000; Gallagher et al., 2002; Sanfey et al., 2003; Harris and Fiske, 2006; Krach et al., 2008; Demoulin et al., 2009; Chaminade et al., 2010; Spunt et al., 2015; Suzuki et al., 2015). Although further studies are necessary to determine the constraints under which robot agents activate the empathizing and mentalizing networks, the aforementioned studies provide preliminary evidence that activation in social brain areas involved in higher-order social-cognitive processes like empathizing and mentalizing (i.e., mPFC, TPJ, insula) more strongly depends on mind perception than activation in social brain areas involved in lower-level social cognitive processing like action understanding (i.e., APS). In particular, it was shown that the APS can reach levels of activation during human–robot interaction that are similar to human–human interaction if certain constraints are met (i.e., human appearance and motor kinematics), while a comparable effect has not been reported for areas like the mPFC, TPJ or insula (i.e., areas get activated by robot agents but to a lesser degree than by human agents). Interestingly, these neuroscientific findings are in line with behavioral studies showing that humans seem to be willing to treat robots as entities with agency (i.e., ability to plan and act), but are reluctant to perceive them as entities that can experience internal states (i.e., ability to sense and feel; Gray et al., 2007). In consequence, research in social robotics would benefit from identifying conditions under which artificial agents engage mechanisms of higher-order social cognition in the human brain, which may necessitate some effort to specifically design robots as intentional and empathetic agents (Gonsior et al., 2012; Silva et al., 2016).

## Effects of Mind Perception on Attitudes and Performance in HRI

Mind perception is not only essential for triggering activation in social brain areas; it also has an impact on how we think and feel about others, and how we perform actions with them (see Waytz et al., 2010b; for a review). These effects on social interactions are mainly positive: mind perception enhances the degree of social connection felt towards others, leads to more prosocial behaviors, motivates others to adhere to moral standards, and improves performance on joint action tasks (see “Positive Effects of Mind Perception in Social Interactions”). Under some circumstances, however, mind perception can be disadvantageous in social interactions, in particular, when the mind status of an agent is ambiguous and evokes categorical uncertainty (i.e., ambiguity regarding whether to classify the agent as human or robot), or when an agent’s behavior deviates strongly enough from human behavior so that an anthropomorphic model would lead to incorrect predictions (see “Negative Effects of Mind Perception in Social Interactions”).

### Positive Effects of Mind Perception in Social Interactions

Treating others as agents with a mind makes us feel socially connected with them and fosters prosocial behaviors, such as decreased cheating and increased generosity (Bering and Johnson, 2005; Haley and Fessler, 2005; Gray et al., 2007, 2012; Shariff and Norenzayan, 2007; Epley et al., 2008). The effect of perceiving a mind in others on prosociality is so strong that simply presenting a pair of eyes during task execution or asking participants to perform a task in front of an audience significantly decreases cheating behaviors and motivates people to perpetuate moral standards (Haley and Fessler, 2005). The positive effect of mind perception on prosocial behavior is even stronger when the interaction partner is similar to the perceiver or believed to belong to his ingroup (Shariff and Norenzayan, 2007; Graham and Haidt, 2010). Agents not being perceived as having a mind, on the other hand, are perceived as being incapable of experiencing emotional states, which makes them unlikely recipients of empathy, morality or prosociality (Haslam, 2006; Hein et al., 2010; Cikara et al., 2011; Harris and Fiske, 2011; Gutsell and Inzlicht, 2012), and makes people feel less guilty when performing harmful acts toward them (Castano and Giner-Sorolla, 2006; Cehajic et al., 2009).

Mind perception also determines whether moral rights are granted to others and how strongly they are judged when showing immoral or harmful behaviors. According to Gray et al. (2007), agents that have a high ability to experience internal and external states, but a low ability to manipulate the environment (i.e., babies or puppies) are treated as ‘moral patients’ who deserve protection, are granted moral rights, and are associated with accidental rather than intentional negative behavior. Agents that display a high degree of agency, but only a low degree of experience (i.e., robots or corporations) are labeled as ‘moral agents’ with full moral responsibilities and the ability to show intentional behavior, in particular when it is harmful. Moral patients are seen as subservient or animalistic, and are more likely to be oppressed against their will or robbed of their human rights (Fiske et al., 2002, 2007), while moral agents are perceived as cold and robotic, and are more likely to be harmed by others (Fiske et al., 2002, 2007; Loughnan and Haslam, 2007). In consequence, this means that in order to be respected as a moral patient, deserving of protection and moral rights, AND as a moral agent, capable of showing intentional behavior, agents need to be ascribed the ability to experience and act. However, while human agents have this set of features by default, robots are typically associated with a limited capability to sense themselves, others and their environments (i.e., reduced ability to experience), with the consequence that they are more likely to be denied moral rights and judged more harshly for behaviors that lead to negative consequences (Gray et al., 2007). This can potentially be prevented by designing robots whose physical and behavioral features trigger mind perception with a high likelihood (e.g., the robot Leonardo; Breazeal et al., 2005).

**FIGURE 3:**
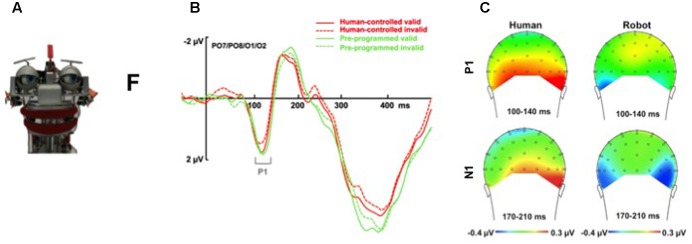
Effect of mind perception on the social relevance of observed behavior. **(A)** Participants are asked to perform a joint attention task (react as fast and accurately as possible to the identity of a target letter: “F” vs. “T”) with the robot EDDIE (designed by Technical University of Munich). Results show that changes in gaze direction are followed more strongly when the eye movements are believed to be intentional versus pre-programmed. **(B)** Grand average ERP waveforms time-locked to the onset of the target for the pool of O1/O2/PO7/PO8 electrodes show that the belief that eye movements are intentional enhances sensory gain control mechanisms, with larger P1 validity effects (i.e., difference between valid and invalid trials) for changes in gaze direction that are believed to be intentional (i.e., human-controlled, red lines) versus non-intentional (i.e., pre-programmed, green lines). **(C)** Topographical maps of voltage distribution (posterior view) show that observing an intentional agent (left panel) versus a pre-programmed agent (right panel) modulates mechanisms of joint attention in occipital and parietal areas (suggesting that attribution of mental states affects visual and early attentional processes). The time interval of the P1 component (100–140 ms) is presented in the upper panel. The time interval of the N1 component (170–210 ms) is presented in the lower panel. For more details see: Wykowska et al. (2014b).

**FIGURE 4:**
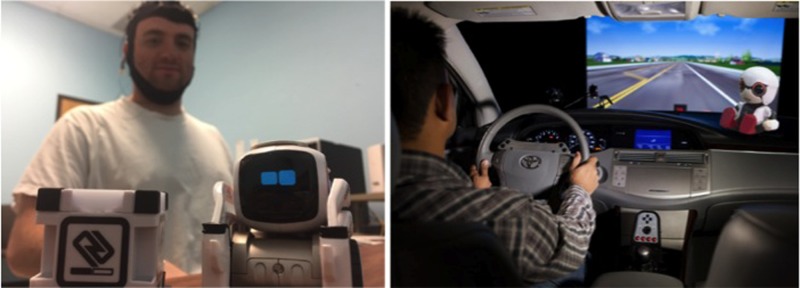
Social facilitation effects in Human–Robot Interaction. Perceiving robot agents as having a mind can induce social facilitation effects on human performance (i.e., presence of a robot agent facilitates performance on simple tasks, but worsens performance on difficult tasks) and foster learning via social reinforcement (i.e., robot provides social cues like smiling for wanted behaviors). The facilitating and reinforcing abilities of companion robots can be used in the classroom to improve learning (left image) or during driving to verbally and non-verbally encourage wanted driving behaviors (right image), for example. Written informed consent has been obtained for publication of the identifiable image on the left. The image on the right was modified (the original image of the driving simulator was retrieved from http://stevevolk.com; the original image of the robot was retrieved from: http://newsroom.toyota.co.jp/).

Believing that an agent has a mind has also been shown to increase the social relevance ascribed to its actions, which can improve performance during social interactions: participants, for instance, follow the eye movements of an agent more strongly when they are believed to reflect intentional compared to preprogrammed or random behavior (Wiese et al., 2012; Wykowska et al., 2014b; Caruana et al., 2016; Özdem et al., 2016; see **Figure 3**). Similarly, perceiving the actions of others as intentional determines how intensely we experience their outcomes (Barrett, 2004; Gilbert et al., 2004): an electric shock hurts more when it is believed to be administered on purpose rather than accidentally (Gray and Wegner, 2008), and intentional harms are judged more rigorously than accidental ones (Ohtsubo, 2007; Cushman, 2008). Perceiving human features like ‘having a mind’ in non-human agents has also been shown to induce social facilitation effects on human performance (Bartneck, 2003; cf. Hoyt et al., 2003; Woods et al., 2005; Park and Catrambone, 2007; Zanbaka et al., 2007; Riether et al., 2012; Hertz and Wiese, 2017), and to foster learning via social reinforcement (Druin and Hendler, 2000; Robins et al., 2005; see **Figure 4**). The facilitatory effect of the presence of an intentional robot on performance becomes even more prominent with an increasing degree of physical embodiment of the robot (Bartneck, 2003; Hoyt et al., 2003; Zanbaka et al., 2007).

### Negative Effects of Mind Perception in Social Interactions

Automatically perceiving mind or human-likeness in non-human agents can also have negative consequences, in particular when an agent is hard to categorize as human versus non-human (Cheetham et al., 2011, 2014; Hackel et al., 2014), or when the anthropomorphic model is not the best predictor for agent behavior (Epley et al., 2007). With regard to categorization difficulties, psychological research has shown that perceiving humanness in others follows a categorical pattern, with agents either being treated as ‘human’ or ‘non-human’ based on their physical features, except at the category boundary located at around 63% of physical humanness, where humanness ratings are ambiguous (Looser and Wheatley, 2010; Cheetham et al., 2011, 2014; Hackel et al., 2014; Martini et al., 2016). The consequence is that pairs of stimuli straddling the category boundary are easier to discriminate (i.e., same or different stimuli?), but harder to categorize (i.e., human or non-human?) than equally similar stimulus pairs located on the same side of the boundary (Repp, 1984; Harnad, 1987; Goldstone and Hendrickson, 2010; Looser and Wheatley, 2010; Cheetham et al., 2011, 2014). Categorizing agents located at the human–nonhuman category boundary results in increased response times and decreased accuracy rates (Cheetham et al., 2011, 2014), consistent with cognitive conflict processing. Trying to resolve this cognitive conflict takes up cognitive resources and can therefore have detrimental effects on performance during tasks that are conjointly performed with agents with an ambiguous mind status (Mandell et al., 2017; Weis and Wiese, 2017).

**FIGURE 5:**
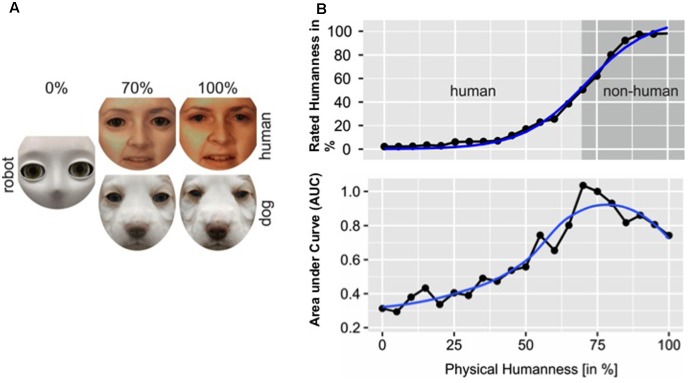
Mind perception can induce a cognitive conflict for agents with ambiguous physical appearance. **(A)** Effects of mind perception triggered by physical appearance can be measured using a morphing procedure (e.g., image of a robot is morphed into image of a human or a dog in steps of 5%). **(B)** Mind perception follows a qualitative (i.e., significant changes in mind ratings occur only after a critical level of physical humanness is reached) rather than a quantitative pattern (i.e., likelihood for perceiving mind increases in a linear fashion with physical humanness). The significant change in mind ratings occurs when the category boundary between human and non-human is crossed (upper panel). Agents located at the category boundary are ambiguous in terms of their mind status, and trying to categorize them as human versus non-human causes a cognitive conflict, which takes cognitive resources to resolve. The degree of cognitive conflict that is induced by a categorical decision can be measured using mouse-tracking (i.e., the more curved the mouse movement, the larger the cognitive conflict; see Freeman and Ambady, 2010). For more details regarding the experiment on cognitive conflict in HRI: Weis and Wiese (2017).

The categorization conflict at the human–nonhuman boundary has also been associated with the uncanny valley phenomenon, where positive attitudes toward non-human agents initially increase as the agents’ physical humanness increases and then drop dramatically as the agents start to look human-*like* but not perfectly human (i.e., *uncanny valley*), just to recover and reach a maximum for agents that are fully human (Mori, 1970; Kätsyri et al., 2015). In particular, it was argued that negative affective reactions associated with uncanny stimuli could be the result of conflict resolution processes triggered by categorical ambiguity during categorization response selection (Cheetham et al., 2011; Burleigh et al., 2013; Kätsyri et al., 2015; see **Figure 5**). Alternatively, perceptions of uncanniness could also be due to a mismatch of agent features, where one feature, for instance physical appearance, suggests that the agent might be human, but another feature, for instance lack of biological motion, suggests otherwise (i.e., *perceptual mismatch*; Seyama and Nagayama, 2007; MacDorman et al., 2009; Mitchell et al., 2011; Saygin et al., 2012; Kätsyri et al., 2015). Both the categorical ambiguity and the perceptual mismatch hypothesis are based on the assumption that physical agent features drive the automatic selection of a neural model that can be used to predict agent behavior, and that categorical ambiguity of the agent or perceptual mismatch of its features can lead to the selection of an inaccurate neural model, which is associated with error processing and might therefore trigger negative affective reactions (Saygin et al., 2012)^4^.

For human–robot interaction, this means that designs should be avoided that (a) trigger categorization difficulties due to physical ambiguity, or (b) cause perceptual mismatch by incorporating human- and machine-like features into the same robot platform. The research also suggests that in order to predict internal states and behaviors of non-human agents, humans need to be able to flexibly activate, correct and apply anthropomorphic knowledge to come up with the best possible prediction given the current circumstances (Epley et al., 2007): when interacting with unfamiliar or novel systems, it makes sense to activate anthropomorphic knowledge and use it as a basis to predict how the agent thinks, feels and behaves. However, as specific agent knowledge becomes available with more experience, the anthropomorphic model needs to be adjusted to match the agent’s actual capabilities (Gilbert, 1991; Gilbert and Malone, 1995), even more so when precise predictions of behavior are required or when future interactions with the agent are anticipated (Epley et al., 2007). Robots that can trigger both an anthropomorphic and a mechanistic mental model also have the advantage that humans can switch between these models depending on their current need for social contact and affiliation or effective task performance. In consequence, this means that robot design should not only focus on mind perception and associated processes of mentalizing and empathizing, but should also equip robots with triggers that activate machine mental models in situations where the anthropomorphic model could potentially lead to incorrect predictions. For example, if a robot cannot grasp an object to pass it to the human user due to hardware or software limitations, it would be useful for the user to understand the underlying reasons so he/she does not blame the robot for a lack of good intentions. This can be achieved, for example, through the use of informative verbal messages (Lee et al., 2010).

## Designing Robots As Intentional Agents

In this section, we explore a number of studies privileging models of the components of the social brain tested in real robots, in real-time human–robot interaction. In doing so, we survey some of the engineering work and technological limitations related to the implementation of interactive robots.

In designing intentional agents, we need to consider the appearance of a robot as well as its behavior (Tapus and Matarić, 2006; Waytz et al., 2010b; Wykowska et al., 2016). Robot appearance is concerned with the ‘bodyware’ or hardware of the machine, while behavior concerns the observable results of the workings of its ‘mindware’ or software. In advanced robot designs, there is a tighter link between the body- and mindware, since often what can be done and how depends on the joint design of hardware and software. Engineering approaches do not necessarily reflect solutions that have any resemblance to their natural counterparts although there is a tradition of robotic research that utilized neuroscience studies as a starting point (Kawato, 1999; Scassellati, 2001; Demiris et al., 2014). Although these approaches led to accurate models of muscular-skeletal systems (Mizuuchi et al., 2006; Pfeifer et al., 2012), facial features (Oh et al., 2006; Becker-Asano et al., 2010), and human kinematics (Kaneko et al., 2009; Metta et al., 2010), they are limited in their ability to reproduce movements accurately in all possible contexts due to technological limitations impacting the range and speed of motion (i.e., mechanics of rigid bodies connected through rotary joints). Furthermore, when talking about mindware, an important distinction needs to be made between neurally accurate models – often proof of principles – and actual working implementations on real hardware, with profound differences between computers and human brains impeding accurate real-time neural simulations of large brain systems, such as those of the social brain. This, however, does not necessarily influence focused experiments targeting specific mechanisms of social-cognitive processing, such as action understanding (via APS) or intention and emotion understanding (via TPJ, mPFC and insula; Oztop et al., 2013).

To build robots that are perceived as intentional agents, we need to ask whether it is even necessary that they accurately emulate human behavior or whether it is sufficient for them to just display certain aspects of human behavior that are most strongly associated with the perception of intentionality (Yamaoka et al., 2007). Given the technological limitations associated with trying to reproduce large brain networks in artificial agents, the goal needs to be the identification of a minimal set of features that can reliably trigger mind perception in non-human agents. Neuroscientists need to identify these features and investigate their effects on attitudes and performance in human–robot interactions, while engineers can help with designing the robot body structure in such a way that faithfully implements this minimal set of behavioral parameters in term of kinematics, dynamics, electronics, and computation. As a corollary to this question, trying to build robots that are perceived as intentional agents can also help to elucidate whether the minimal set of parameters relates to a specific architecture and how tuning various parameters affects the way a robot is perceived.

From an engineering perspective, research in robotics and artificial intelligence that may have an impact to intentionality is vast (see Feil-Seifer and Matarić, 2009; for a review). First attempts to build socially competent robots can be traced back to the MIT robots Kismet (Breazeal, 2003) and Cog (Brooks et al., 1999). With Kismet, Breazeal and Scassellati (1999) studied how an expressive robot elicited appropriate social responses in humans by displaying attention and turn-taking mechanisms. They also identify some of the requirements of the visual system of such robots (Breazeal et al., 2001) as for example the advantages of foveated vision, eye contact (and therefore detecting the eyes of the interactant in the visual scene), and a number of sensorimotor control loops (e.g., avoid and seek objects and people). Scassellati (2002) went further and took some first steps toward implementing a theory of mind for the robot Cog based on an established psychological model for mentalizing developed by Baron-Cohen (1997). Among other features, the model possesses a human-like attentional system that identifies living agents and non-living objects from basic perceptual features like optical flow. In particular, the model relies on an Intentionality Detector (ID) that labels actions as intentional based on their goal-directedness, as well as an Eye Direction Detector (EDD) that allows the robot to shift its attention to locations in space that are gazed-at by its human interaction partner. Although the ID on Cog was relatively simple, dealing exclusively with the issue of animacy versus no animacy, it nevertheless had the advantage of being based on a psychologically sound and empirically derived model of mentalization.

Following the discovery of mirror neurons in non-human primates and their involvement in action understanding (Gallese et al., 1996), neuroscientifically inspired approaches to robotics mainly focused on developing models for action recognition and imitation (Metta et al., 2006; Oztop et al., 2013). The key concept of shared sensorimotor representations, dating back to Liberman and Mattingly (1985), guided a variety of implementations utilizing, for example, recurrent neural networks (Tani et al., 2004) or various other machine-learning methods that learn direct-inverse models from examples (Oztop et al., 2006; Demiris, 2007). Among these attempts to implement a mirror neuron system into artificial agents, some models were more neuroscientifically accurate than others (Arbib et al., 2000; see Oztop et al., 2013; for a review). More recently, the use of RGBd cameras boosted the ability to extract meaningful parameters automatically from images allowing robots to engage in more complex social interactions with their human counterparts. The use of convolutional neural networks made a further step toward robust body pose/skeleton extraction from images (even 2D; see Cao et al., 2016), which is a fundamental component for robots to interact in a complex way within a social context.

More recently, Pointeau et al. (2013) utilized both object recognition and human posture detection to give a humanoid robot the ability to implement spatial perspective taking during the execution of a shared task in human–robot interaction. Spatial reasoning was implemented via simulation of the environment in 3D, which allowed for disambiguating linguistic constructs (e.g., ‘object on the left’). An autobiographical memory was utilized to learn the structure of the shared task, which was represented as a sequence of elemental steps allowing the robot to take the human’s perspective and to step in at any given point of the task execution. Although this architecture bears some resemblance with certain brain functions, such as memorizing sequences and spatial perspective taking, its implementation still relies exclusively on engineering methods, utilizing simple tables and strictly symbolic representations, instead of neurologically plausible mechanisms.

Other areas of research relevant to the design of robots as intentional agents include image and object recognition, as well as spatial reasoning. In terms of object recognition, brain-inspired models have dominated the field for several years (Serre et al., 2005), but are being replaced by the modern “brute force” approach of using very large neural networks and managing the increased computational cost through specialized processors (e.g., GPUs), resulting in an improvement in performance of orders of magnitude (Krizhevsky et al., 2012). In terms of spatial navigation, roboticists have developed a set of standard methods including probabilistic localization techniques and planning impact-free movements (O’Donnell and Lozano-Pérez, 1989; Thrun, 2002), some of which are also building blocks for robot controllers that help avoid contact and/or reach properly during human–robot interaction (Kulić and Croft, 2005;De Santis et al., 2008). Other active research directions within the theme of spatial reasoning explore how to represent spatial data (i.e., objects and people in 3D, their spatial relationships), and how to connect linguistic constructs that imply spatial relationships with reasoning (Sugiyama et al., 2006; Gold et al., 2009; Hato et al., 2010). Spatial knowledge is one element of the correct interpretation of deictic gestures, which by their nature require both the gesture itself and general knowledge about the environment, which usually, in human–human interaction, co-occur with utterances. Therefore, for the robot to understand them, location and recognition of the hand configuration, the spatial configuration of objects/people in the world, and speech recognition have to be integrated (Brooks and Breazeal, 2006).

In summary, this short overview indicates that some of the problems in designing intentional robots require competencies that span the whole range of human cognitive skills in both perceptual and reasoning terms, and that psychologically and neuroscientifically sound implementations thereof are for the most part missing. Furthermore, while important aspects of human–robot interaction are currently addressed in isolated models, a more integrated architecture that combines cognitive and social functioning does not exist and the effectiveness of the existing models on mind perception and attitudes and performance in human–robot interaction has not been sufficiently investigated. In the future, neuroscientists and roboticists need to work together to identify at least a minimal set of physical and behavioral robot features that have the potential to activate the same areas in the human brain as human interaction partners. In doing so, it is still not guaranteed that the exact functioning of the human neural system can be emulated in artificial agents, but it at least increases the likelihood that robot agents are treated *as if* they were intentional agents.

## Conclusion

We highlight that the design of social robots should be based on methods of cognitive neuroscience in order to determine robot features (e.g., behavioral features, such as timing of saccades, head-eye coordination, frequency and length of gaze toward a human user) that activate mechanisms of social cognition in the human brain. Neuroscientific results inform us about what these mechanisms are, how they are implemented in human neural architecture and when they are activated. These results can also inspire research in artificial intelligence and robotics so that robot architectures can be based on similar principles as those operating in the human brain (even if this is at present often a challenging enterprise due to technological limitations), and allow for more human-like behaviors of robots. As one of the key factors activating mechanisms of social cognition is attribution of intentionality to robots, it is important to understand the conditions under which humans perceive robots as intentional agents, and what consequences attribution of intentionality may have for human–robot interaction. Although adopting an anthropomorphic mental model in explaining the behaviors of robot agents usually has positive consequences on attitudes and performance in human–robot interaction, in some cases it might hinder the quality of human–robot interaction, in particular when some agent features trigger mind perception and others do not. Therefore, it is extremely important to design robots based on systematic studies, perhaps with an iterative approach, in order to understand which parameters of the robot’s behavior and appearance activate the social brain and elicit attribution of intentionality, and whether in certain cases it is better not to evoke mind attribution.

## Author Contributions

EW and AW conceptualized the paper, and created the figures. AW wrote sections “Introduction” and “Conclusion”, EW wrote sections “Can Robots Be Perceived as Intentional Agents?,” “Observing Intentional Agents Activates Social Brain Areas,” “Effects of Mind Perception on Attitudes and Performance in HRI,” and GM wrote section “Designing Robots as Intentional Agents.”

## Conflict of Interest Statement

The authors declare that the research was conducted in the absence of any commercial or financial relationships that could be construed as a potential conflict of interest.

## Footnotes

1 https://www.sony.net/SonyInfo/News/Press_Archive/199806/98-052/

2 Note that we use the term ‘intentionality’ in the philosophical sense: “Intentionality is the power of minds to be about, to represent, or to stand for, things, properties and states of affairs” (Jacob, 2014). In other words, intentionality characterizes mental states to refer to something. For example, a belief (mental state) is *about* something, refers to something. This way of defining intentionality might be different from the common use of the term “intentional” (commonly “intentional” might be understood as “done on purpose” or “deliberate”). The philosophical meaning dates back to Brentano: “Every mental phenomenon is characterized by what the Scholastics of the Middle Ages called the intentional (or mental) inexistence of an object, and what we might call, though not wholly unambiguously, reference to a content, direction toward an object (which is not to be understood here as meaning a thing), or immanent objectivity” (Brentano, 1874, p. 68). We use the term ‘intentional’ in the philosophical sense in order to highlight the aspect of robots being potentially perceived as agents with mental states (as opposed to only mindless machines).

3 Webpages for Siri (https://www.apple.com/ios/siri/), Waymo (https://waymo.com/), Jibo (https://www.jibo.com/).

4 Please note that conflicts might also lead to positive effects in human–robot interaction given that they might lead human interaction partners to update their internal representation of the robot to match better its abilities and features.

## References

[B1] Adolphs R. (2009). The social brain: neural basis of social knowledge. *Annu. Rev. Psychol.* 60 693–716. 10.1146/annurev.psych.60.110707.163514 18771388 PMC2588649

[B2] Allman J. M. Hakeem A. Erwin J. M. Nimchinsky E. Hof P. (2001). The anterior cingulate cortex: the evolution of an interface between emotion and cognition. *Ann. N. Y. Acad. Sci.* 935 107–117. 10.1111/j.1749-6632.2001.tb03476.x 11411161

[B3] Ames D. L. Jenkins A. C. Banaji M. R. Mitchell J. P. (2008). Taking another person’s perspective increases self-referential neural processing. *Psychol. Sci.* 19 642–644. 10.1111/j.1467-9280.2008.02135.x 18727776

[B4] Amodio D. M. Frith C. D. (2006). Meeting of minds: the medial frontal cortex and social cognition. *Nat. Rev. Neurosci.* 7 268–277. 10.1038/nrn1884 16552413

[B5] Anzalone S. M. Tilmont E. Boucenna S. Xavier J. Jouen A.-L. Bodeau N. (2014). How children with autism spectrum disorder behave and explore the 4-dimensional (spatial 3D+ time) environment during a joint attention induction task with a robot. *Res. Autism Spectr. Disord.* 8 814–826. 10.1016/j.rasd.2014.03.002

[B6] Arbib M. A. Billard A. Iacoboni M. Oztop E. (2000). Synthetic brain imaging: grasping, mirror neurons and imitation. *Neural Netw.* 13 975–997. 10.1016/S0893-6080(00)00070-8 11156205

[B7] Balas B. Tonsager C. (2014). Face animacy is not all in the eyes: evidence from contrast chimeras. *Perception* 43 355–367. 10.1068/p7696 25109004

[B8] Barch D. M. Braver T. S. Akbudak E. Conturo T. Ollinger J. Snyder A. (2001). Anterior cingulate cortex and response conflict: effects of response modality and processing domain. *Cereb. Cortex* 11 837–848. 10.1093/cercor/11.9.837 11532889

[B9] Baron-Cohen S. (1997). *Mindblindness: An Essay on Autism and Theory of Mind.* Boston, MA: MIT Press.

[B10] Baron-Cohen S. (2005). “The empathizing system: a revision of the 1994 model of the mindreading system,” in *Origins of the Social Mind*, eds Ellis B. Bjorklund D. (New York City, NY: Guilford Publications).

[B11] Baron-Cohen S. Leslie A. M. Frith U. (1985). Does the autistic child have a theory of mind? *Cognition* 21 37–46. 10.1016/0010-0277(85)90022-8 2934210

[B12] Barrett J. L. (2004). *Why Would Anyone Believe in God?.* Lanham, MD: AltaMira Press.

[B13] Bartneck C. (2003). “Interacting with an embodied emotional character,” in *Proceedings of the 2003 International Conference on Designing Pleasurable Products and Interfaces*, DPPI, Pittsburgh, PA, 55–60. 10.1145/782896.782911

[B14] Bartneck C. Reichenbach J. (2005). Subtle emotional expressions of synthetic characters. *Int. J. Hum. Comput. Stud.* 62 179–192. 10.1016/j.ijhcs.2004.11.006

[B15] Basteris A. Nijenhuis S. M. Stienen A. H. Buurke J. H. Prange G. B. Amirabdollahian F. (2014). Training modalities in robot-mediated upper limb rehabilitation in stroke: a framework for classification based on a systematic review. *J. Neuroeng. Rehabil.* 11:111 10.1186/1743-0003-11-111 PMC4108977 25012864

[B16] Bastian B. Haslam N. (2010). Excluded from humanity: the dehumanizing effects of social ostracism. *J. Exp.Soc. Psychol.* 46 107–113. 10.1016/j.jesp.2009.06.022

[B17] Becchio C. Adenzato M. Bara B. (2006). How the brain understands intention: different neural circuits identify the componential features of motor and prior intentions. *Conscious. Cogn.* 15 64–74. 10.1016/j.concog.2005.03.006 15935699

[B18] Becker-Asano C. Ogawa K. Nishio S. Ishiguro H. (2010). “Exploring the uncanny valley with Geminoid HI-1 in a real-world application,” in *Proceedings of IADIS International Conference Interfaces and Human Computer Interaction*, Freiburg, 121–128.

[B19] Bekele E. Crittendon J. A. Swanson A. Sarkar N. Warren Z. E. (2014). Pilot clinical application of an adaptive robotic system for young children with autism. *Autism* 18 598–608. 10.1177/1362361313479454 24104517 PMC3980197

[B20] Bering J. M. Johnson D. D. P. (2005). O Lord, you perceive my thoughts from afar: recursiveness and the evolution of supernatural agency. *J. Cogn. Cult.* 5 118–141. 10.1163/1568537054068679

[B21] Birks M. Bodak M. Barlas J. Harwood J. Pether M. (2016). Robotic seals as therapeutic tools in an aged care facility: a qualitative study. *J. Aging Res.* 2016 1–7. 10.1155/2016/8569602 PMC5136401 27990301

[B22] Bisio A. Sciutti A. Nori F. Metta G. Fadiga L. Sandini G. (2014). Motor contagion during human-human and human-robot interaction. *PLOS ONE* 9:e106172 10.1371/journal.pone.0106172 PMC4143359 25153990

[B23] Blakemore S.-J. Decety J. (2001). From the perception of action to the understanding of intention. *Nat. Rev. Neurosci.* 2 561–567. 10.1038/35086023 11483999

[B24] Brass M. Derrfuss J. von Cramon D. Y. (2005). The inhibition of imitative and overlearned responses: a functional double dissociation. *Neuropsychologia* 43 89–98. 10.1016/j.neuropsychologia.2004.06.018 15488909

[B25] Brass M. Schmitt R. Spengler S. Gergely G. (2007). Investigating action understanding: inferential processes versus action simulation. *Curr. Biol.* 17 2117–2121. 10.1016/j.cub.2007.11.057 18083518

[B26] Breazeal C. (2003). Toward sociable robots. *Rob. Auton. Syst.* 42 167–175. 10.1016/S0921-8890(02)00373-1

[B27] Breazeal C. Buchsbaum D. Gray J. Gatenby D. Blumberg B. (2005). Learning from and about others: towards using imitation to bootstrap the social understanding of others by robots. *Artif. Life* 11 31–62. 10.1162/1064546053278955 15811219

[B28] Breazeal C. Edsinger A. Fitzpatrick P. Scassellati B. (2001). Active vision for sociable robots. *IEEE Trans. Syst. Man Cybern. Syst.* 31 443–453. 10.1109/3468.952718

[B29] Breazeal C. Scassellati B. (1999). “How to build robots that make friends and influence people,” in *Proceedings of the IEEE/RSJ International Conference on Intelligent Robots and Systems IROS’99* Vol. 2 Kyoto, 858–863. 10.1109/IROS.1999.812787

[B30] Brentano F. (1874). *Psychology from an Empirical Standpoint*, trans. Rancurello A. C. Terrell D. B. McAlister L. L. London: Routledge.

[B31] Brooks A. G. Breazeal C. (2006). “Working with robots and objects: revisiting deictic reference for achieving spatial common ground,” in *Proceedings of the 1st ACM SIGCHI/SIGART Conference on Human-Robot Interaction*, (New York City, NY: Association for Computing Machinery), 297–304. 10.1145/1121241.1121292

[B32] Brooks R. A. Breazeal C. Marjanović M. Scassellati B. Williamson M. M. (1999). “The cog project: building a humanoid robot,” in *Computation for Metaphors, Analogy, and Agents*, ed. Nehaniv C. L. (Berlin: Springer), 52–87.

[B33] Brothers L. (2002). “The social brain: a project for integrating primate behavior and neurophysiology in a new domain,” in *Foundations in Social Neuroscience*, ed. Cacioppo J. T. (London: MIT Press), 367–385.

[B34] Buccino G. Binkofski F. Fink G. R. Fadiga L. Fogassi L. Gallese V. (2001). Action observation activates premotor and parietal areas in a somatotopic manner: an fMRI study. *Eur. J. Neurosci.* 13 400–404. 10.1111/j.1460-9568.2001.01385.x 11168545

[B35] Burleigh T. J. Schoenherr J. R. Lacroix G. L. (2013). Does the uncanny valley exist? An empirical test of the relationship between eeriness and the human likeness of digitally created faces. *Comput. Hum. Behav.* 29 759–771. 10.1016/j.chb.2012.11.021

[B36] Bush G. Luu P. Posner M. I. (2000). Cognitive and emotional influences in anterior cingulate cortex. *Trends Cogn. Sci.* 4 215–222. 10.1016/S1364-6613(00)01483-2 10827444

[B37] Cabibihan J. J. Javed H. Ang M. Jr. Aljunied S. M. (2013). Why robots? a survey on the roles and benefits of social robots in the therapy of children with autism. *Int. J. Soc. Robot.* 5 593–618. 10.1007/s12369-013-0202-2

[B38] Cao Z. Simon T. Wei S. E. Sheikh Y. (2016). Realtime Multi-Person 2D pose estimation using part affinity fields. arXiv:1611.08050v2 10.1109/TPAMI.2019.2929257 31331883

[B39] Caruana N. McArthur G. Woolgar A. Brock J. (2016). Simulating social interactions for the experimental investigation of joint attention. *Neurosci. Biobehav. Rev.* 74 115–125. 10.1016/j.neubiorev.2016.12.022 28027954

[B40] Castano E. Giner-Sorolla R. (2006). Not quite human: infrahumanization in response to collective responsibility for intergroup killing. *J. Pers. Soc. Psychol.* 90 804–818. 10.1037/0022-3514.90.5.804 16737374

[B41] Castelli F. Happé F. Frith U. Frith C. (2000). Movement and mind: a functional imaging study of perception and interpretation of complex intentional movement patterns. *Neuroimage* 12 314–325. 10.1006/nimg.2000.0612 10944414

[B42] Castledine A. R. Chalmers C. (2011). LEGO robotics: an authentic problem solving tool? *Des. Technol. Educ.* 16 19–27.

[B43] Cavanna A. E. Trimble M. R. (2006). The precuneus: a review of its functional anatomy and behavioural correlates. *Brain* 129 564–583. 10.1093/brain/awl004 16399806

[B44] Cehajic S. Brown R. Gonzalez R. (2009). What do I care? Perception of ingroup responsibility and dehumanization as predictors of empathy felt for the victim group. *Group Process. Intergroup Relat.* 12 715–729. 10.1177/1368430209347727

[B45] Chaminade T. Cheng G. (2009). Social cognitive neuroscience and humanoid robotics. *J. Physiol. Paris* 103 286–295. 10.1016/j.jphysparis.2009.08.011 19665550

[B46] Chaminade T. Decety J. (2002). Leader or follower? Involvement of the inferior parietal lobule in agency. *Neuroreport* 13 1975–1978. 10.1097/00001756-200210280-00029 12395103

[B47] Chaminade T. Hodgins J. Kawato M. (2007). Anthropomorphism influences perception of computer-animated character’s actions. *Soc. Cogn. Affect. Neurosci.* 2 206–216. 10.1093/scan/nsm017 18985142 PMC2569803

[B48] Chaminade T. Rosset D. Da Fonseca D. Nazarian B. Lutcher E. Cheng G. (2012). How do we think machines think? An fMRI study of alleged competition with an artificial intelligence. *Front. Hum. Neurosci.* 6:103 10.3389/fnhum.2012.00103 PMC3347624 22586381

[B49] Chaminade T. Zecca M. Blakemore S. J. Takanishi A. Frith C. D. Micera S. (2010). Brain response to a humanoid robot in areas implicated in the perception of human emotional gestures. *PLOS ONE* 5:e11577 10.1371/journal.pone.0011577 PMC2908128 20657777

[B50] Chang C. Lee J. Chao P. Wang C. Chen G. (2010). Exploring the possibility of using humanoid robots as instructional tools for teaching a second language in primary school. *Educ. Technol. Soc.* 13 13–24.

[B51] Chang C. F. Hsu T. Y. Tseng P. Liang W. K. Tzeng O. J. Hung D. L. (2013). Right temporoparietal junction and attentional reorienting. *Hum. Brain Mapp.* 34 869–877. 10.1002/hbm.21476 22419442 PMC6870429

[B52] Cheetham M. Suter P. Jancke L. (2011). The human likeness dimension of the “uncanny valley hypothesis”: behavioral and functional MRI findings. *Front. Hum. Neurosci.* 5:126 10.3389/fnhum.2011.00126 PMC3223398 22131970

[B53] Cheetham M. Suter P. Jancke L. (2014). Perceptual discrimination difficulty and familiarity in the uncanny valley: more like a “Happy Valley.”. *Front. Psychol.* 5:1219 10.3389/fpsyg.2014.01219 PMC4237038 25477829

[B54] Chong T. T. Cunnington R. Williams M. A. Kanwisher N. Mattingley J. B. (2008). fMRI adaptation reveals mirror neurons in human inferior parietal cortex. *Curr. Biol.* 18 1576–1580. 10.1016/j.cub.2008.08.068 18948009 PMC2766090

[B55] Church W. Ford T. Perova N. Rogers C. (2010). “Physics with robotics: using Lego Mindstorms in high school education,” in *Proceedings of Advancement of Artificial Intelligence Spring Symposium*, Stanford, CA, 47–49.

[B56] Cikara M. Bruneau J. L. Saxe S. T. (2011). From agents to objects: sexist attitudes and neural responses to sexualized targets. *J. Cogn. Neurosci.* 23 540–551. 10.1162/jocn.2010.21497 20350187 PMC3801174

[B57] Cortellessa G. Scopelliti M. Tiberio L. Koch Svedberg G. Loutfi A. Pecora F. (2008). “A cross-cultural evaluation of domestic assistive robots,” in *Proceedings of the AAAI Fall Symposium on AI in Eldercare: New Solutions to Old Problems*, Arlington, VA.

[B58] Costa A. Torriero S. Oliveri M. Caltagirone C. (2008). Prefrontal and temporo-parietal involvement in taking others’ perspective: TMS evidence. *Behav. Neurol.* 19 71–74. 10.1155/2008/694632 18413921 PMC5452444

[B59] Critchley H. D. Mathias C. J. Josephs O. O’Doherty J. Zanini S. Dewar B. K. (2003). Human cingulate cortex and autonomic control: converging neuroimaging and clinical evidence. *Brain* 126 2139–2152. 10.1093/brain/awg216 12821513

[B60] Cross E. S. Liepelt R. Hamilton A. F. C. Parkinson J. Ramsey R. Stadler W. (2011). Robotic movement preferentially engages the action observation network. *Hum. Brain Mapp.* 33 2238–2254. 10.1002/hbm.21361 21898675 PMC6870135

[B61] Cullen H. Ryota K. Bahrami B. Rees G. (2014). Individual differences in anthropomorphic attributions and human brain structure. *Soc. Cogn. Affect. Neurosci.* 9 1276–1280. 10.1093/scan/nst109 23887807 PMC4158361

[B62] Cushman F. (2008). Crime and punishment: distinguishing the roles of causal and intentional analyses in moral judgment. *Cognition* 108 353–380. 10.1016/j.cognition.2008.03.006 18439575

[B63] Dautenhahn K. (2003). Roles and functions of robots in human society: implications from research in autism therapy. *Robotica* 21 443–452. 10.1017/S0263574703004922

[B64] de Guzman M. Bird G. Banissy M. J. Catmur C. (2016). Self–other control processes in social cognition: from imitation to empathy. *Philos. Trans. R. Soc. B Sci.* 371 20150079 10.1098/rstb.2015.0079 PMC4685524 26644597

[B65] De Santis A. Siciliano B. De Luca A. Bicchi A. (2008). An atlas of physical human–robot interaction. *Mech. Mach. Theory* 43 253–270. 10.1016/j.mechmachtheory.2007.03.003

[B66] Decety J. Chaminade T. (2003). When the self represents the other: a new cognitive neuroscience view on psychological identification. *Consci. Cogn.* 12 577–596. 10.1016/S1053-8100(03)00076-X 14656502

[B67] Decety J. Grèzes J. (1999). Neural mechanisms subserving the perception of human actions. *Trends Cogn. Sci.* 3 172–178. 10.1016/S1364-6613(99)01312-1 10322473

[B68] Demiris Y. (2007). Prediction of intent in robotics and multi-agent systems. *Cogn. Process.* 8 151–158. 10.1007/s10339-007-0168-9 17479306

[B69] Demiris Y. Aziz-Zadeh L. Bonaiuto J. (2014). Information processing in the mirror neuron system in primates and machines. *Neuroinformatics* 12 63–91. 10.1007/s12021-013-9200-7 24085487

[B70] Demoulin S. Cortes B. P. Viki T. G. Rodriguez A. P. Rodriguez R. T. Paladino M. P. (2009). The role of ingroup identification in infra-humanization. *Int. J. Psychol.* 44 4–11. 10.1080/00207590802057654 22029436

[B71] Deska J. C. Almaraz S. M. Hugenberg K. (2016). Of mannequins and men: ascriptions of mind in faces are bounded by perceptual and processing similarities to human faces. *Soc. Psychol. Pers. Sci.* 8 183–190. 10.1177/1948550616671404

[B72] Dinstein I. Hasson U. Rubin N. Heeger D. J. (2007). Brain areas selective for both observed and executed movements. *J. Neurophysiol.* 98 1415–1427. 10.1152/jn.00238.2007 17596409 PMC2538553

[B73] Druin A. Hendler J. A. (2000). *Robots for Kids: Exploring New Technologies for Learning.* San Francisco, CA: Morgan Kaufmann.

[B74] Epley N. Waytz A. Akalis S. Cacioppo J. T. (2008). When I need a human: motivational determinants of anthropomorphism. *Soc. Cogn.* 26 143–155. 10.1521/soco.2008.26.2.143

[B75] Epley N. Caruso E. M. (2009). “Perspective taking: misstepping into others’ shoes,” in *Handbook of Imagination and Mental Simulation*, eds Markman K. D. Klein W. M. P. Suhr J. A. (New York, NY: Psychology Press). 10.4135/9781412958479.n397

[B76] Epley N. Morewedge C. K. Keysar B. (2004). Perspective taking in children and adults: equivalent egocentrism but differential correction. *J. Exp. Soc. Psychol.* 40 760–768. 10.1016/j.jesp.2004.02.002

[B77] Epley N. Waytz A. Cacioppo J. T. (2007). On seeing human: a three-factor theory of anthropomorphism. *Psychol. Rev.* 114 864–886. 10.1037/0033-295X.114.4.864 17907867

[B78] Farrer C. Franck N. Georgieff N. Frith C. D. Decety J. Jeannerod M. (2003). Modulating the experience of agency: a positron emission tomography study. *Neuroimage* 18 324–333. 10.1016/S1053-8119(02)00041-1 12595186

[B79] Feil-Seifer D. Matarić M. J. (2009). “Human robot interaction,” in *Encyclopedia of Complexity and Systems Science*, ed. Meyers R. A. (New York, NY: Springer), 4643–4659.

[B80] Fernandes E. Fermé E. Oliveira R. (2006). “Using robots to learn function in math class,” in *Proceedings of the ICMI 17 Study Conference*, eds Son L. H. Sinclair N. Lagrange J. B. Hoyles C. (Hanoi: Hanoi University of Technology).

[B81] Fiske S. T. Cuddy A. J. C. Glick P. (2007). Universal dimensions of social cognition: warmth and competence. *Trends Cogn. Sci.* 11 77–83. 10.1016/j.tics.2006.11.005 17188552

[B82] Fiske S. T. Cuddy A. J. C. Glick P. Xu J. (2002). A model of (often mixed) stereotype content: competence and warmth respectively follow from perceived status and competition. *J. Pers. Soc. Psychol.* 82 878–902. 10.1037/0022-3514.82.6.878 12051578

[B83] Flandorfer P. (2012). Population ageing and socially assistive robots for elderly persons: the importance of sociodemographic factors for user acceptance. *Int. J. Popul. Res.* 2012 13 10.1155/2012/829835

[B84] Freeman J. B. Ambady N. (2010). MouseTracker: software for studying real-time mental processing using a computer mouse-tracking method. *Behav. Res. Methods* 42 226–241. 10.3758/BRM.42.1.226 20160302

[B85] Friesen C. K. Kingstone A. (1998). The eyes have it! Reflexive orienting is triggered by nonpredictive gaze. *Psychon. Bull. Rev.* 5 490–495. 10.3758/BF03208827

[B86] Frith C. D. Frith U. (2006a). How we predict what other people are going to do. *Brain Res.* 1079 36–46. 10.1016/j.brainres.2005.12.126 16513098

[B87] Frith C. D. Frith U. (2006b). The neural basis of mentalizing. *Neuron* 50 531–534. 10.1016/j.neuron.2006.05.001 16701204

[B88] Frith U. Frith C. D. (2001). The biological basis of social interaction. *Curr. Dir. Psychol. Sci.* 10 151–155. 10.1111/1467-8721.00137

[B89] Frith U. Frith C. D. (2003). Development and neurophysiology of mentalizing. *Philos. Trans. R. Soc. Lond. B Biol. Sci.* 358 459–473. 10.1098/rstb.2002.1218 12689373 PMC1693139

[B90] Fujita M. Kitano H. (1998). Development of an autonomous quadruped robot for robot entertainment. *Auton. Agent.* 5 7–18. 10.1007/978-1-4615-5735-7_2

[B91] Gallagher H. L. Frith C. D. (2003). Functional imaging of “theory of mind.”. *Trends Cogn. Sci.* 7 77–83. 10.1016/S1364-6613(02)00025-6 12584026

[B92] Gallagher H. L. Happé F. Brunswick N. Fletcher P. C. Frith U. Frith C. D. (2000). Reading the mind in cartoons and stories: an fMRI study of ‘theory of mind’ in verbal and nonverbal tasks. *Neuropsychologia* 38 11–21. 10.1016/S0028-3932(99)00053-6 10617288

[B93] Gallagher H. L. Jack A. Roepstorff A. Frith C. (2002). Imaging the intentional stance in a competitive game. *Neuroimage* 16:814 10.1006/nimg.2002.1117 12169265

[B94] Gallese V. Fadiga L. Fogassi L. Rizzolatti G. (1996). Action recognition in the premotor cortex. *Brain* 119 593–609. 10.1093/brain/119.2.593 8800951

[B95] Gallese V. Keysers C. Rizzolatti G. (2004). A unifying view of the basis of social cognition. *Trends Cogn. Sci.* 8 396–403. 10.1016/j.tics.2004.07.002 15350240

[B96] Gao T. McCarthy G. Scholl B. J. (2010). The wolfpack effect perception of animacy irresistibly influences interactive behavior. *Psychol. Sci.* 21 1845–1853. 10.1177/0956797610388814 21078895

[B97] Gazzola V. Rizzolatti G. Wicker B. Keysers C. (2007). The anthropomorphic brain: the mirror neuron system responds to human and robotic actions. *Neuroimage* 35 1674–1684. 10.1016/j.neuroimage.2007.02.003 17395490

[B98] Gilbert D. T. (1991). How mental systems believe. *Am. Psychol.* 46 107–119. 10.1037/0003-066X.46.2.107

[B99] Gilbert D. T. Lieberman M. D. Morewedge C. K. Wilson T. D. (2004). The peculiar longevity of things not so bad. *Psychol. Sci.* 15 14–19. 10.1111/j.0963-7214.2004.01501003.x 14717826

[B100] Gilbert D. T. Malone P. S. (1995). The correspondence bias. *Psychol. Bull.* 117 21–38. 10.1037/0033-2909.117.1.21 7870861

[B101] Gold K. Doniec M. Crick C. Scassellati B. (2009). Robotic vocabulary building using extension inference and implicit contrast. *Artif. Intell.* 173 145–166. 10.1016/j.artint.2008.09.002

[B102] Goldstone R. L. Hendrickson A. T. (2010). Categorical perception. *Cogn. Sci.* 1 69–78. 10.1002/wcs.26 26272840

[B103] Gonsior B. Sosnowski S. Buß M. Wollherr D. Kühnlenz K. (2012). “An emotional adaption approach to increase helpfulness towards a robot,” in *Intelligent Robots and Systems (IROS), 2012 IEEE/RSJ International Conference*, Vilamoura, 2429–2436. 10.1109/IROS.2012.6385941

[B104] Gould S. J. (1996). Can we truly know sloth and rapacity? *Nat. Hist.* 105 18–57.

[B105] Graf B. Parlitz C. Hägele M. (2009). “Robotic home assistant care-O-bot 3 product vision and innovation platform,” in *Human-Computer Interaction. Novel Interaction Methods and Techniques. HCI 2009. Lecture Notes in Computer Science*, ed. Jacko J. A. (Berlin: Springer), 312–320. 10.1007/978-3-642-02577-8_34

[B106] Grafton S. T. Hamilton A. F. (2007). Evidence for a distributed hierarchy of action representation in the brain. *Hum. Mov. Sci.* 26 590–616. 10.1016/j.humov.2007.05.009 17706312 PMC2042582

[B107] Graham J. Haidt J. (2010). Beyond beliefs: religions bind individuals into moral communities. *Pers. Soc. Psychol. Rev.* 14 140–150. 10.1177/1088868309353415 20089848

[B108] Gray H. M. Gray K. Wegner D. M. (2007). Dimensions of mind perception. *Science* 315 619 10.1126/science.1134475 17272713

[B109] Gray K. Wegner D. M. (2008). The sting of intentional pain. *Psychol. Sci.* 19 1260–1262. 10.1111/j.1467-9280.2008.02208.x 19121135

[B110] Gray K. Young L. Waytz A. (2012). Mind perception is the essence of morality. *Psychol. Inq.* 23 101–124. 10.1080/1047840X.2012.651387 22754268 PMC3379786

[B111] Grèzes J. Berthoz S. Passingham R. E. (2006). Amygdala activation when one is the target for deceit: Did he lie to you or someone else? *Neuroimage* 30 601–608. 10.1016/j.neuroimage.2005.09.038 16257239

[B112] Grèzes J. Frith C. Passingham R. E. (2004). Brain mechanisms for inferring deceit in the actions of others. *J. Neurosci.* 24 5500–5505. 10.1523/JNEUROSCI.0219-04.2004 15201322 PMC6729335

[B113] Gutsell J. N. Inzlicht M. (2012). Intergroup differences in the sharing of emotive states: neural evidence of an empathy gap. *Soc. Cogn. Affect. Neurosci.* 7 596–603. 10.1093/scan/nsr035 21705345 PMC3375887

[B114] Hackel L. M. Looser C. E. Van Bavel J. J. (2014). Group membership alters the threshold for mind perception: the role of social identity, collective identification, and intergroup threat. *J. Exp. Soc. Psychol.* 52 15–23. 10.1016/j.jesp.2013.12.001

[B115] Haley K. J. Fessler D. M. T. (2005). Nobody’s watching? Subtle cues affect generosity in an anonymous economic game. *Evol. Hum. Behav.* 26 245–256. 10.1016/j.evolhumbehav.2005.01.002

[B116] Harnad S. (1987). “Psychophysical and cognitive aspects of categorical perception: a critical overview,” in *Categorical Perception: The Groundwork of Cognition*, ed. Harnad S. (New York, NY: Cambridge University Press).

[B117] Harris L. T. Fiske S. T. (2006). Dehumanizing the lowest of the low – neuroimaging responses to extreme out-groups. *Psychol. Sci.* 17 847–853. 10.1111/j.1467-9280.2006.01793.x 17100784

[B118] Harris L. T. Fiske S. T. (2011). “Perceiving humanity or not: a social neuroscience approach to dehumanized perception,” in *Social Neuroscience: Toward Understanding the Underpinnings of the Social Mind*, eds Todorov A. Fiske S. T. Prentice D. A. (New York, NY: Oxford University Press), 123–134.

[B119] Haslam N. (2006). Dehumanization: an integrative review. *Pers. Soc. Psychol. Rev.* 10 252–264. 10.1207/s15327957pspr1003-4 16859440

[B120] Hato Y. Satake S. Kanda T. Imai M. Hagita N. (2010). “Pointing to space: modeling of deictic interaction referring to regions,” in *5th ACM/IEEE International Conference on Human-Robot Interaction (HRI)*, Boca Raton, FL, 301–308. 10.1145/1734454.1734559

[B121] Hein G. Silani G. Preuschoff K. Batson C. D. Singer T. (2010). Neural responses to ingroup and outgroup members’ suffering predict individual differences in costly helping. *Neuron* 68 149–160. 10.1016/j.neuron.2010.09.003 20920798

[B122] Hertz N. Wiese E. (2017). “Social facilitation with nonhuman agents: possible or not?,” in *Proceedings of HFES 2017*, Austin, TX.

[B123] Hinds P. Roberts T. Jones H. (2004). Whose job is it anyway? A study of human–robot interaction in a collaborative task. *Hum. Comput. Interact.* 19 151–181. 10.1207/s15327051hci1901&2_7

[B124] Hogan N. Krebs H. I. (2004). Interactive robots for neuro-rehabilitation. *Restor. Neurol. Neurosci.* 22 349–358. 15502275

[B125] Hoyt C. L. Blascovich J. Swinth K. R. (2003). Social inhibition in immersive virtual environments. *Presence* 12 183–195. 10.1162/105474603321640932

[B126] Huey E. D. Krueger F. Grafman J. (2006). Representations in the human prefrontal cortex. *Curr. Dir. Psychol. Sci.* 15 167–171. 10.1111/j.1467-8721.2006.00429.x

[B127] Hynes C. A. Baird A. A. Grafton S. T. (2006). Differential role of the orbital frontal lobe in emotional versus cognitive perspective-taking. *Neuropsychologia* 44 374–383. 10.1016/j.neuropsychologia.2005.06.011 16112148

[B128] Iacoboni M. (2005). Neural mechanisms of imitation. *Curr. Opin. Neurobiol.* 15 632–637. 10.1016/j.conb.2005.10.010 16271461

[B129] Jackson P. L. Brunet E. Meltzoff A. N. Decety J. (2006). Empathy examined through the neural mechanisms involved in imagining how I feel versus how you would feel pain: an event-related fMRI study. *Neuropsychologia* 44 752–761. 10.1016/j.neuropsychologia.2005.07.015 16140345

[B130] Jacob P. (2014). “Intentionality,” in *The Stanford Encyclopedia of Philosophy*, ed. Zalta E. Stanford, CA: Stanford University.

[B131] Johnson M. Demiris Y. (2005). Perceptual perspective taking and action recognition. *Int. J. Adv. Robot. Syst.* 2 32 10.5772/5775

[B132] Kajopoulos J. Wong A. H. Y. Yuen A. W. C. Dung T. A. Tan Y. K. Wykowska A. (2015). “Robot-assisted training of joint attention skills in children diagnosed with autism,” in *Lecture Notes in Artificial Intelligence*, eds Randy G. Yuzuru T. Wolfgang W. (Berlin: Springer), 296–305. 10.1007/978-3-319-25554-5-30

[B133] Kaneko K. Kanehiro F. Morisawa M. Miura K. Nakaoka S. I. Kajita S. (2009). “Cybernetic human HRP-4C,” in *Proceedings of the 9th IEEE-RAS International Conference on Humanoid Robots, Humanoids*, Paris, 7–14. 10.1109/ICHR.2009.5379537

[B134] Kaplan F. (2004). Who is afraid of the humanoid? Investigating cultural differences in the acceptance of robots. *Int. J. HR* 1 465–480. 10.1142/S0219843604000289

[B135] Kätsyri J. Förger K. Mäkäräinen M. Takala T. (2015). A review of empirical evidence on different uncanny valley hypotheses: support for perceptual mismatch as one road to the valley of eeriness. *Front. Psychol.* 6:390 10.3389/fpsyg.2015.00390 PMC4392592 25914661

[B136] Kawato M. (1999). Internal models for motor control and trajectory planning. *Curr. Opin. Neurobiol.* 9 718–727. 10.1016/S0959-4388(99)00028-8 10607637

[B137] Keysers C. Perrett D. I. (2004). Demystifying social cognition: a Hebbian perspective. *Trends Cogn. Sci.* 8 501–507. 10.1016/j.tics.2004.09.005 15491904

[B138] Keysers C. Wicker B. Gazzola V. Anton J. L. Fogassi L. Gallese V. (2004). A touching sight: SII/PV activation during the observation and experience of touch. *Neuron* 42 335–346. 10.1016/S0896-6273(04)00156-4 15091347

[B139] Kidd C. D. Breazeal C. (2008). “Robots at home: understanding long-term human-robot interaction,” in *Proceedings of the IEEE/RSJ International Conference on Intelligent Robots and Systems (IROS)*, Nice, 3230–3235. 10.1109/IROS.2008.4651113

[B140] Kilner J. M. Neal A. Weiskopf N. Friston K. J. Frith C. D. (2009). Evidence of mirror neurons in human inferior frontal gyrus. *J. Neurosci.* 29 10153–10159. 10.1523/JNEUROSCI.2668-09.2009 19675249 PMC2788150

[B141] Kilner J. M. Paulignan Y. Blakemore S. J. (2003). An interference effect of observed biological movement on action. *Curr. Biol.* 13 522–525. 10.1016/S0960-9822(03)00165-9 12646137

[B142] Knapp M. L. Hall J. A. Horgan T. G. (2013). *Nonverbal Communication in Human Interaction*, 8th Edn Belmont, CA: Wadsworth Publishing.

[B143] Knoblich G. Jordan J. S. (2003). Action coordination in groups and individuals: learning anticipatory control. *J. Exp. Psychol.* 29 1006–1016. 10.1037/0278-7393.29.5.1006 14516231

[B144] Kory J. Breazeal C. (2014). “Storytelling with robots: learning companions for preschool children’s language development,” in *Proceedings of the 23rd IEEE International Symposium on Robot and Human Interactive Communication (RO-MAN)*, eds Vargas P. A. Aylett R. (Washington, DC: IEEE), 10.1109/ROMAN.2014.6926325

[B145] Kozima H. Nakagawa C. (2007). “A robot in a playroom with preschool children: longitudinal field practice,” in *Proceedings of the 16th IEEE International Symposium on Robot and Human interactive Communication (RO-MAN)*, New York, NY, 1058–1059. 10.1109/ROMAN.2007.4415238

[B146] Krach S. Hegel F. Wrede B. Sagerer G. Binkofski F. Kircher T. (2008). Can machines think? Interaction and perspective taking with robots investigated via fMRI. *PLOS ONE* 3:e2597 10.1371/journal.pone.0002597 PMC2440351 18612463

[B147] Krall S. C. Rottschy C. Oberwelland E. Bzdok D. Fox P. T. Eickhoff S. B. (2015). The role of the right temporoparietal junction in attention and social interaction as revealed by ALE meta-analysis. *Brain Struct. Funct.* 220 587–604. 10.1007/s00429-014-0803-z 24915964 PMC4791048

[B148] Krall S. C. Volz L. J. Oberwelland E. Grefkes C. Fink G. R. Konrad K. (2016). The right temporoparietal junction in attention and social interaction: A transcranial magnetic stimulation study. *Hum. Brain Mapp.* 37 796–807. 10.1002/hbm.23068 26610283 PMC6867405

[B149] Krizhevsky A. Sutskever I. Hinton G. E. (2012). “Imagenet classification with deep convolutional neural networks,” in *Proceedings of the 25th International Conference on Advances in Neural Information Processing Systems*, Lake Tahoe, NV, 1097–1105.

[B150] Kulić D. Croft E. A. (2005). Safe planning for human-robot interaction. *J. Field Robot.* 22 383–396. 10.1002/rob.20073

[B151] Kupferberg A. Huber M. Helfer B. Lenz C. Knoll A. Glasauer S. (2012). Moving just like you: motor interference depends on similar motility of agent and observer. *PLOS ONE* 7:e39637 10.1371/journal.pone.0039637 PMC3384618 22761853

[B152] Lee M. K. Kiesler S. Forlizzi J. Srinivasa S. Rybski P. (2010). “Gracefully mitigating breakdowns in robotic services,” in *Proceedings of the ACM/IEEE International Conference on Human-Robot Interaction (HRI)*, Pittsburgh, PA, 203–210.

[B153] Leslie A. M. Friedman O. German T. P. (2004). Core mechanisms in theory of mind. *Trends Cogn. Sci.* 8 528–533. 10.1016/j.tics.2004.10.001 15556021

[B154] Leyens J. P. Paladino P. M. Rodriguez-Torres R. Vaes J. Demoulin S. Rodriguez-Perez A. (2000). The emotional side of prejudice: the attribution of secondary emotions to ingroups and outgroups. *Pers. Soc. Psychol. Rev.* 4 186–197. 10.1207/S15327957PSPR0402_06

[B155] Liberman A. M. Mattingly I. G. (1985). The motor theory of speech perception revised. *Cognition* 21 1–36. 10.1016/0010-0277(85)90021-6 4075760

[B156] Looser C. E. Guntupalli J. S. Wheatley T. (2013). Multivoxel patterns in face-sensitive temporal regions reveal an encoding schema based on detecting life in a face. *Soc. Cogn. Affect. Neurosci.* 8 799–805. 10.1093/scan/nss078 22798395 PMC3791074

[B157] Looser C. E. Wheatley T. (2010). The tipping point of animacy: how, when, and where we perceive life in a face. *Psychol. Sci.* 21 1854–1862. 10.1177/0956797610388044 21097720

[B158] Loughnan S. Haslam N. (2007). Animals and androids: implicit associations between social categories and nonhumans. *Psychol. Sci.* 18 116–121. 10.1111/j.1467-9280.2007.01858.x 17425529

[B159] MacDorman K. F. Green R. D. Ho C.-C. Koch C. T. (2009). Too real for comfort? Uncanny responses to computer generated faces. *Comput. Hum. Behav.* 25 695–710. 10.1016/j.chb.2008.12.026 PMC4264966 25506126

[B160] Mandell A. Smith M. Wiese E. (2017). “Mind perception in humanoid agents has negative effects on cognitive processing,” in *Proceedings of Human Factors and Ergonomics Society*, Austin, TX.

[B161] Martin R. F. Carlos A. D. Jose Maria C. P. Gonzalo A. D. Raul B. M. Rivero S. (2013). Robots in therapy for dementia patients. *J. Phys. Agents* 7 49–56.

[B162] Martini M. C. Gonzalez C. A. Wiese E. (2016). Seeing minds in others–Can agents with robotic appearance have human-like preferences? *PLOS ONE* 11:e0146310 10.1371/journal.pone.0146310 PMC4706415 26745500

[B163] Maurer D. Le Grand R. Mondloch C. J. (2002). The many faces of configural processing. *Trends Cogn. Sci.* 6 255–260. 10.1016/S1364-6613(02)01903-4 12039607

[B164] McCabe K. Houser D. Ryan L. Smith V. Trouard T. A. (2001). A functional imaging study of cooperation in two person reciprocal exchange. *Proc. Natl. Acad. Sci. U.S.A.* 98 11832–11835. 10.1073/pnas.211415698 11562505 PMC58817

[B165] Metta G. Natale L. Nori F. Sandini G. Vernon D. Fadiga L. (2010). The iCub humanoid robot: an open-systems platform for research in cognitive development. *Neural Netw.* 23 1125–1134. 10.1016/j.neunet.2010.08.010 20864311

[B166] Metta G. Sandini G. Natale L. Craighero L. Fadiga L. (2006). Understanding mirror neurons: a bio-robotic approach. *Interact. Stud.* 7 197–232. 10.1075/is.7.2.06met

[B167] Metta G. Sandini G. Vernon D. Natale L. Nori F. (2008). “The iCub humanoid robot: an open platform for research in embodied cognition,” in *Proceedings of the 8th Workshop on Performance Metrics for Intelligent Systems*, Gaithersburg, MD.

[B168] Mitchell J. P. (2008). Activity in right temporo-parietal junction is not selective for theory-of-mind. *Cereb. Cortex* 18 262–271. 10.1093/cercor/bhm051 17551089

[B169] Mitchell J. P. Macrae C. N. Banaji M. R. (2006). Dissociable medial prefrontal contributions to judgments of similar and dissimilar others. *Neuron* 50 655–663. 10.1016/j.neuron.2006.03.040 16701214

[B170] Mitchell W. J. Szerszen K. A. Lu A. S. Schermerhorn P. W. Scheutz M. MacDorman K. F. (2011). A mismatch in the human realism of face and voice produces an uncanny valley. *Iperception* 2 10–12. 10.1068/i0415 23145223 PMC3485769

[B171] Mizuuchi I. Yoshikai T. Sodeyama Y. Nakanishi Y. Miyadera A. Yamamoto T. (2006). “Development of musculoskeletal humanoid kotaro,” in *Proceedings of The 2006 IEEE International Conference on Robotics and Automation*, Orlando, FL.

[B172] Moore C. Dunham P. (1995). *Joint Attention: Its Origins and Role in Development.* Mahwah, NJ: Lawrence Erlbaum Associates.

[B173] Mori M. (1970). Bukimi no tani [the uncanny valley]. *Energy* 7 33–35.

[B174] Mubin O. Stevens C. J. Shadid S. Al Mahmud A. Dong J. J. (2013). A review of the applicability of robots in education. *Technol. Educ. Learn.* 1 1–7. 10.2316/Journal.209.2013.1.209-0015

[B175] Mukamel R. Ekstrom A. Kaplan J. Iacoboni M. Fried I. (2010). Single neuron responses in humans during execution and observation of actions. *Curr. Biol.* 20 750–756. 10.1016/j.cub.2010.02.045 20381353 PMC2904852

[B176] Nagel T. (1974). What is it like to be a bat? *Philos. Rev.* 83 435–450. 10.1017/CBO9781107341050.014

[B177] Neven L. (2010). ‘But obviously not for me’: robots, laboratories and the defiant identity of elder test users. *Sociol. Health Illn.* 32 335–347. 10.1111/j.1467-9566.2009.01218.x 20149151

[B178] Nomura M. Iidaka T. Kakehi K. Tsukiura T. Hasegawa T. Maeda Y. (2003). Frontal lobe networks for effective processing of ambiguously expressed emotions in humans. *Neurosci. Lett.* 348 113–116. 10.1016/S0304-3940(03)00768-7 12902031

[B179] Oberman L. M. McCleery J. P. Ramachandran V. S. Pineda J. A. (2007). EEG evidence for mirror neuron activity during the observation of human and robot actions: toward an analysis of the human qualities of interactive robots. *Neurocomputing* 70 2194–2203. 10.1016/j.neucom.2006.02.024

[B180] O’Donnell P. A. Lozano-Pérez T. (1989). “Deadlock-free and collision-free coordination of two robot manipulators,” in *IEEE International Conference on Robotics and Automation* Vol. 89 Cambridge, MA, 484–489. 10.1109/ROBOT.1989.100033

[B181] Oh J. H. Hanson D. Kim W. S. Han Y. Kim J. Y. Park I. W. (2006). “Design of android type humanoid robot Albert HUBO,” in *Proceedings of the IEEE/RSJ International Conference on Intelligent Robots and Systems*, Secaucus, NJ, 1428–1433. 10.1109/IROS.2006.281935

[B182] Ohnishi T. Moriguchi Y. Matsuda H. Mori T. Hirakata M. Imabayashi E. (2004). The neural network for the mirror system and mentalizing in normally developed children: an fMRI study. *Neuroreport* 15 1483–1487. 10.1097/01.wnr.0000127464.17770.1f 15194879

[B183] Ohtsubo Y. (2007). Perceiver intentionality intensifies blameworthiness of negative behaviors: blame-praise asymmetry in intensification effect. *J. Psychol. Res.* 49 100–110. 10.1111/j.1468-5884.2007.00337.x

[B184] Özdem C. Wiese E. Wykowska A. Müller H. Brass M. Van Overwalle F. (2016). Believing androids–fMRI activation in the right temporo-parietal junction is modulated by ascribing intentions to non-human agents. *Soc. Neurosci.* 12 582–593. 10.1080/17470919.2016.1207702 27391213

[B185] Oztop E. Franklin D. Chaminade T. Gordon C. (2005). Human-humanoid interaction: is a humanoid robot perceived as a human. *Int. J. HR* 2 537–559. 10.1142/S0219843605000582

[B186] Oztop E. Kawato M. Arbib M. (2006). Mirror neurons and imitation: a computationally guided review. *Neural Netw.* 19 254–271. 10.1016/j.neunet.2006.02.002 16595172

[B187] Oztop E. Kawato M. Arbib M. A. (2013). Mirror neurons: functions, mechanisms and models. *Neurosci. Lett.* 540 43–55. 10.1016/j.neulet.2012.10.005 23063951

[B188] Park S. Catrambone R. (2007). Social facilitation effects of virtual humans. *Hum. Factors* 49 1054–1060. 10.1518/001872007X249910 18074704

[B189] Perner J. Aichhorn M. Kronbichler M. Staffen W. Ladurner G. (2006). Thinking of mental and other representations: the roles of left and right temporo-parietal junction. *Soc. Neurosci.* 1 245–258. 10.1080/17470910600989896 18633791

[B190] Pfeifer R. Lungarella M. Iida F. (2012). The challenges ahead for bio-inspired ’soft‘ robotics. *Commun. ACM* 55 76–87. 10.1145/2366316.2366335

[B191] Pobric G. Hamilton A. (2006). Action understanding requires the left inferior frontal cortex. *Curr. Biol.* 16 524–529. 10.1016/j.cub.2006.01.033 16527749

[B192] Pointeau G. Petit M. Ford Dominey P. (2013). “Embodied simulation based on autobiographical memory,” in *Living Machines*, eds Lepora N. F. Mura A. Krapp H. G. Verschure P. F. M. J. Prescott T. J. (Berlin: Springer-Verlag), 240–250. 10.1007/978-3-642-39802-5-21

[B193] Prange G. B. Jannink M. J. A. Groothuis-Oudshoorn C. G. M. Hermens H. J. IJzerman M. J. (2006). Systematic review of the effect of robot-aided therapy on recovery of the hemiparetic arm after stroke. *J. Rehabil. Res. Dev.* 43 171–184. 10.1682/JRRD.2005.04.0076 16847784

[B194] Press C. Bird G. Flach R. Heyes C. (2005). Robotic movement elicits automatic imitation. *Cogn. Brain Res.* 25 632–640. 10.1016/j.cogbrainres.2005.08.020 16344220

[B195] Press C. Gillmeister H. Heyes C. (2006). Bottom-up, not top-down, modulation of imitation by human and robotic models. *Eur. J. Neurosci.* 24: 2415–2419. 10.1111/j.1460-9568.2006.05115.x 17042792

[B196] Press C. Gillmeister H. Heyes C. (2007). Sensorimotor experience enhances automatic imitation of robotic action. *Proc. R. Soc. B* 274 2509–2514. 10.1098/rspb.2007.0774 PMC2275887 17698489

[B197] Preston S. D. de Waal F. B. M. (2002). Empathy: its ultimate and proximate bases. *Behav. Brain Sci.* 25 1–72. 10.1017/S0140525X02000018 12625087

[B198] Repp B. H. (1984). “Categorical perception: issues, methods, findings,” in *Speech and Language: Advances in Basic Research and Practice* Vol. 10 ed. Lass J. (New York, NY: Academic Press), 243–335. 10.1016/B978-0-12-608610-2.50012-1

[B199] Ricks D. J. Colton M. B. (2010). “Trends and considerations in robot assisted autism therapy,” in *Proceedings of the IEEE International Conference on Robotics and Automation (ICRA)*, Anchorage, AK, 4354–4359. 10.1109/ROBOT.2010.5509327

[B200] Riedl R. Mohr P. Kenning P. Davis F. D. Heekeren H. (2014). Trusting humans and avatars: a brain imaging study based on evolution theory. *J. Manage. Inf. Syst.* 30 83–113. 10.2753/MIS0742-1222300404

[B201] Riether N. Hegel F. Wrede B. Horstmann G. (2012). “Social facilitation with social robots?,” in *Proceedings of the Seventh Annual ACM/IEEE International Conference on Human-Robot Interaction*, Boston, MA, 41–47. 10.1145/2157689.2157697

[B202] Rizzolatti G. Craighero L. (2004). The mirror-neuron system. *Annu. Rev. Neurosci.* 27 169–192. 10.1146/annurev.neuro.27.070203.144230 15217330

[B203] Robins B. Dautenhahn K. Te Boekhorst R. Billard A. (2005). Robotic assistants in therapy and education of children with autism: can a small humanoid robot help encourage social interaction skills? *Univers. Access Inf. Soc.* 4 105–120. 10.1007/s10209-005-0116-3

[B204] Rosset E. (2008). It’s no accident: our bias for intentional explanations. *Cognition* 108 771–780. 10.1016/j.cognition.2008.07.001 18692779

[B205] Ruby P. Decety J. (2001). Effect of subjective perspective taking during simulation of action: a PET investigation of agency. *Nat. Neurosci.* 4 546–550. 11319565 10.1038/87510

[B206] Samson D. Apperly I. A. Braithwaite J. J. Andrews B. J. Bodley Scott S. E. (2010). Seeing it their way: Evidence for rapid and involuntary computation of what other people see. *J. Exp. Psychol.* 36 1255–1266. 10.1037/a0018729 20731512

[B207] Samson D. Apperly I. A. Chiavarino C. Humphreys G. W. (2004). Left temporo-parietal junction is necessary for representing someone else’s belief. *Nat. Neurosci.* 7 499–500. 10.1038/nn1223 15077111

[B208] Sanfey A. G. Rilling J. K. Aronson J. A. Nystrom L. E. Cohen J. D. (2003). The neural basis of economic decision-making in the ultimatum game. *Science* 300 1755–1758. 10.1126/science.1082976 12805551

[B209] Saxe R. Kanwisher N. (2003). People thinking about thinking people: the role of the temporo-parietal junction in “theory of mind.”. *Neuroimage* 19 1835–1842. 10.1016/S1053-8119(03)00230-1 12948738

[B210] Saxe R. Powell L. J. (2006). It’s the thought that counts: specific brain regions for one component of theory of mind. *Psychol. Sci.* 17 692–699. 10.1111/j.1467-9280.2006.01768.x 16913952

[B211] Saxe R. Wexler A. (2005). Making sense of another mind: the role of the right temporo-parietal junction. *Neuropsychologia* 43 1391–1399. 10.1016/j.neuropsychologia.2005.02.013 15936784

[B212] Saygin A. P. (2007). Superior temporal and premotor brain areas necessary for biological motion perception. *Brain* 130 2452–2461. 10.1093/brain/awm162 17660183

[B213] Saygin A. P. Chaminade T. Ishiguro H. Driver J. Frith C. (2012). The thing that should not be: predictive coding and the uncanny valley in perceiving human and humanoid robot actions. *Soc. Cogn. Affect. Neurosci.* 7 413–422. 10.1093/scan/nsr025 21515639 PMC3324571

[B214] Saygin A. P. Wilson S. M. Hagler D. J. Jr. Bates E. Sereno M. I. (2004). Point-light biological motion perception activates human premotor cortex. *J. Neurosci.* 24 6181–6188. 10.1523/JNEUROSCI.0504-04.2004 15240810 PMC6729669

[B215] Scassellati B. (2001). *Foundations for a Theory of Mind for a Humanoid Robot*, Ph.D. thesis, Massachusetts Institute of Technology, Cambridge, MA.

[B216] Scassellati B. (2002). Theory of mind for a humanoid robot. *Auton. Robots* 12 13–24. 10.1037/e445252005-001

[B217] Scassellati B. Admoni H. Matarić M. (2012). Robots for use in autism research. *Annu. Rev. Biomed. Eng.* 14 275–294. 10.1146/annurev-bioeng-071811-150036 22577778

[B218] Schein C. Gray K. (2015). The unifying moral dyad liberals and conservatives share the same harm-based moral template. *Pers. Soc. Psychol. Bull.* 41 1147–1163. 10.1177/0146167215591501 26091912

[B219] Scholz J. Triantafyllou C. Whitfield-Gabrieli S. Brown E. N. Saxe R. (2009). Distinct regions of right temporo-parietal junction are selective for theory of mind and exogenous attention. *PLOS ONE* 4:e4869 10.1371/journal.pone.0004869 PMC2653721 19290043

[B220] Scopelliti M. Giuliani M. V. Fornara F. (2005). Robots in a domestic setting: a psychological approach. *Univers. Access Inf. Soc.* 4 146–155. 10.1007/s10209-005-0118-1

[B221] Sebanz N. Knoblich G. (2009). Prediction in joint action: what, when, and where. *Top. Cogn. Sci.* 1 353–367. 10.1111/j.1756-8765.2009.01024 25164938

[B222] Sebanz N. Knoblich G. Prinz W. (2005). How two share a task: corepresenting stimulus-response mappings. *J. Exp. Psychol.* 31 1234–1246. 10.1037/0096-1523.31.6.1234 16366786

[B223] Sebanz N. Knoblich G. Prinz W. Wascher E. (2006). Twin peaks: An ERP study of action planning and control in co-acting individuals. *J. Cogn. Neurosci.* 18 859–870. 10.1162/jocn.2006.18.5.859 16768383

[B224] Serre T. Wolf L. Poggio T. (2005). “Object recognition with features inspired by visual cortex,” in *Proceedings of the IEEE Computer Society Conference on Computer Vision and Pattern Recognition* Vol. 2 Washington, DC, 994–1000. 10.1109/CVPR.2005.254

[B225] Seyama J. Nagayama R. S. (2007). The uncanny valley: effect of realism on the impression of artificial human faces. *Presence* 16 337–351. 10.1162/pres.16.4.337

[B226] Shariff A. F. Norenzayan A. (2007). God is watching you: priming God concepts increases prosocial behavior in an anonymous economic game. *Psychol. Sci.* 18 803–809. 10.1111/j.1467-9280.2007.01983 17760777

[B227] Sharkey N. (2008). The ethical frontiers of robotics. *Science* 322 1800–1801. 10.1126/science.1164582 19095930

[B228] Shibata T. Mitsui T. Wada K. Touda A. Kumasaka T. Tagami K. (2001). “Mental commit robot and its application to therapy of children,” in *Proceedings of IEEE/ASME International Conference on Advanced Intelligent Mechatronics*, Como, 1053–1058. 10.1109/AIM.2001.936838

[B229] Silva R. Louro L. Malheiro T. Erlhagen W. Bicho E. (2016). Combining intention and emotional state inference in a dynamic neural field architecture for human-robot joint action. *Adapt. Behav.* 24 350–372. 10.1177/1059712316665451

[B230] Singer T. (2006). The neuronal basis and ontogeny of empathy and mind reading: review of literature and implications for future research. *Neurosci. Biobehav. Rev.* 30 855–863. 10.1016/j.neubiorev.2006.06.011 16904182

[B231] Singer T. Seymour B. O’Doherty J. Kaube H. Dolan R. J. Frith C. D. (2004). Empathy for pain involves the affective but not sensory components of pain. *Science* 303 1157–1162. 10.1126/science.1093535 14976305

[B232] Spunt R. P. Meyer M. L. Lieberman M. D. (2015). The default mode of human brain function primes the intentional stance. *J. Cogn. Neurosci.* 27 1116–1124. 10.1162/jocn_a_00785 25603027

[B233] Steinbeis N. (2016). The role of self–other distinction in understanding others’ mental and emotional states: neurocognitive mechanisms in children and adults. *Philos. Trans. R. Soc. B Sci.* 371:20150074 10.1098/rstb.2015.0074 PMC4685520 26644593

[B234] Sugiyama O. Kanda T. Imai M. Ishiguro H. Hagita N. (2006). Human-like conversation with gestures and verbal cues based on three-layer attention-drawing model. *Conn. Sci.* 18 379–402. 10.1080/09540090600890254

[B235] Suzuki Y. Galli L. Ikeda A. Itakura S. Kitazaki M. (2015). Measuring empathy for human and robot hand pain using electroencephalography. *Sci. Rep.* 5:15924 10.1038/srep15924 PMC4630641 26525705

[B236] Takayama L. Ju W. Nass C. (2008). “Beyond dirty, dangerous and dull: what everyday people think robots should do,” in *Proceedings of the 3rd ACM/IEEE International Conference on Human Robot Interaction*, Amsterdam, 25–32.

[B237] Tani J. Ito M. Sugita Y. (2004). Self-organization of distributedly represented multiple behavior schemata in a mirror system: reviews of robot experiments using RNNPB. *Neural Netw.* 17 1273–1289. 10.1016/j.neunet.2004.05.007 15555866

[B238] Tapus A. Matarić M. J. (2006). Towards socially assistive robotics. *Int. J. Robot. Soc. Jpn.* 24 576–578. 10.7210/jrsj.24.576

[B239] Tapus A. Mataric M. J. Scasselati B. (2007). Socially assistive robotics [Grand challenges of robotics]. *IEEE Robot. Autom. Mag.* 14 35–42. 10.1109/MRA.2007.339605

[B240] Tapus A. Peca A. Aly A. Pop C. A. Jisa L. Pintea S. (2012). Children with autism social engagement in interaction with Nao, an imitative robot–A series of single case experiments. *Interact. Stud.* 13 315–347. 10.1075/is.13.3.01tap

[B241] Thrun S. (2002). Probabilistic robotics. *Commun. ACM* 45 52–57. 10.1145/504729.504754

[B242] Triebel R. Arras K. Alami R. Beyer L. Breuers S. Chatila R. (2016). “Spencer: a socially aware service robot for passenger guidance and help in busy airports,” in *Field and Service Robotics*, eds Wettergreen D. Barfoot T. (Cham: Springer), 607–622. 10.1007/978-3-319-27702-8_40

[B243] Tversky B. Hard B. M. (2009). Embodied and disembodied cognition: spatial perspective taking. *Cognition* 110 124–129. 10.1016/j.cognition.2008.10.008 19056081

[B244] Umilta M. A. Kohler E. Gallese V. Fogassi L. Fadiga L. Keysers C. (2001). I know what you are doing. A neurophysiological study. *Neuron* 31 155–165. 10.1016/S0896-6273(01)00337-3 11498058

[B245] van Overwalle F. (2009). Social cognition and the brain: a meta-analysis. *Hum. Brain Mapp.* 30 829–858. 10.1002/hbm.20547 18381770 PMC6870808

[B246] van Schie H. T. Mars R. B. Coles M. G. Bekkering H. (2004). Modulation of activity in medial frontal and motor cortices during error observation. *Nat. Neurosci.* 7 549–554. 10.1038/nn1239 15107858

[B247] Vollm B. A. Taylor A. N. Richardson P. Corcoran R. Stirling J. McKie S. (2006). Neuronal correlates of theory of mind and empathy: a functional magnetic resonance imaging study in a nonverbal task. *Neuroimage* 29 90–98. 10.1016/j.neuroimage.2005.07.022 16122944

[B248] Wada K. Shibata T. (2006). “Robot therapy in a care house - its sociopsychological and physiological effects on the residents,” in *Proceedings of IEEE International Conference on Robotics and Automation*, Orlando, FL, 3966–3971.

[B249] Wada K. Shibata T. Musha T. Kimura S. (2005). “Effects of robot therapy for demented patients evaluated by EEG,” in *Proceedings of IEEE/RSJ International Conference on Intelligent Robots and Systems*, Edmonton, AB, 1552–1557.

[B250] Wagner D. D. Kelley W. M. Heatherton T. F. (2011). Individual differences in the spontaneous recruitment of brain regions supporting mental state understanding when viewing natural social scenes. *Cereb. Cortex* 21 2788–2796. 10.1093/cercor/bhr074 21527789 PMC3209798

[B251] Ward S. A. Parikh S. Workman B. (2011). Health perspectives: international epidemiology of ageing. *Best Pract. Res. Clin. Anaesthesiol.* 25 305–317. 10.1016/j.bpa.2011.05.002 21925398

[B252] Warren Z. E. Zheng Z. Swanson A. R. Bekele E. Zhang L. Crittendon J. A. (2015). Can robotic interaction improve joint attention skills? *J. Autism Dev. Dis.* 45 1–9. 10.1007/s10803-013-1918-4 PMC3949684 24014194

[B253] Waytz A. Cacioppo J. Epley N. (2010a). Who sees human? The importance and stability of individual differences in anthropomorphism. *Perspect. Psychol. Sci.* 5 219–232. 10.1177/1745691610369336 24839457 PMC4021380

[B254] Waytz A. Gray K. Epley N. Wegner D. M. (2010b). Causes and consequences of mind perception. *Trends Cogn. Sci.* 14 383–388. 10.1016/j.tics.2010.05.006 20579932

[B255] Weis P. Wiese E. (2017). “Cognitive conflict as possible cause for the uncanny valley,” in *Proceedings of Human Factors and Ergonomics Society*, Santa Monica, CA.

[B256] Wheatley T. Weinberg A. Looser C. Moran T. Hajcak G. (2011). Mind perception: real but not artificial faces sustain neural activity beyond the N170/VPP. *PLOS ONE* 6:e17960 10.1371/journal.pone.0017960 PMC3069036 21483856

[B257] Wicker B. Keysers C. Plailly J. Royet J. P. Gallese V. Rizzolatti G. (2003). Both of us disgusted in my insula: the common neural basis of seeing and feeling disgust. *Neuron* 40 655–664. 10.1016/S0896-6273(03)00679-2 14642287

[B258] Wiese E. Wykowska A. Zwickel J. Müller H. J. (2012). I see what you mean: how attentional selection is shaped by ascribing intentions to others. *PLOS ONE* 7:e45391 10.1371/journal.pone.0045391 PMC3458834 23049794

[B259] Wimmer H. Perner J. (1983). Beliefs about beliefs: representation and constraining function of wrong beliefs in young children’s understanding of deception. *Cognition* 13 103–128. 10.1016/0010-0277(83)90004-5 6681741

[B260] Wood J. N. Grafman J. (2003). Human prefrontal cortex: processing and representational perspectives. *Nat. Rev. Neurosci.* 4 139–147. 10.1038/nrn1033 12563285

[B261] Woods S. Dautenham K. Kaouri C. (2005). “Is someone watching me? – Consideration of social facilitation effects in human-robot interaction experiments,” in *IEEE International Symposium on Computational Intelligence in Robotics and Automation, CIRA*, Espoo, 53–60. 10.1109/CIRA.2005.1554254

[B262] Wykowska A. Chaminade T. Cheng G. (2016). Embodied artificial agents for understanding human social cognition. *Philos. Trans. R. Soc. Lond. B Biol. Sci.* 371:20150375 10.1098/rstb.2015.0375 PMC4843613 27069052

[B263] Wykowska A. Chellali R. Al-Amin M. Md Müller H. J. (2014a). Implications of robot actions for human perception. How do we represent actions of the observed robots?. *Int. J. Soc. Robot.* 6 357–366. 10.1007/s12369-014-0239-x

[B264] Wykowska A. Wiese E. Prosser A. Müller H. J. (2014b). Beliefs about the minds of others influence how we process sensory information. *PLOS ONE* 9:e94339 10.1371/journal.pone.0094339 PMC3979768 24714419

[B265] Yamaoka F. Kanda T. Ishiguro H. Hagita N. (2007). How contingent should a lifelike robot be? The relationship between contingency and complexity. *Conn. Sci.* 19 143–162. 10.1145/1121241.1121294

[B266] Yamazaki R. Christensen L. Skov K. Chang C. Damholdt M. Sumioka H. (2016). Intimacy in phone conversations: anxiety reduction for danish seniors with hugvie. *Front. Psychol.* 7:537 10.3389/fpsyg.2016.00537 PMC4835483 27148144

[B267] Zanbaka C. Ulinski A. Goolkasian P. Hodges L. F. (2007). Social responses to virtual humans: Implications for future interface design. *Proceedings of the SIGCHI Conference on Human Factors in Computing Systems, CHI*, San Jose, CA, 1561–1570. 10.1145/1240624.1240861

[B268] Zink C. F. Kempf L. Hakimi S. Rainey C. A. Stein J. L. Meyer-Lindenberg A. (2011). Vasopressin modulates social recognition-related activity in the left temporoparietal junction in humans. *Trans. Psychiatry* 1:e3 10.1038/tp.2011.2 PMC3309468 22832391

[B269] Złotowski J. Proudfoot D. Yogeeswaran K. Bartneck C. (2015). Anthropomorphism: opportunities and challenges in human–robot interaction. *Int. J. Soc. Robot.* 7 347–360. 10.1007/s12369-014-0267-6

[B270] Zwickel J. (2009). Agency attribution and visuo-spatial perspective taking. *Psychon. Bull. Rev.* 16 1089–1093. 10.3758/PBR.16.6.1089 19966260

